# Aurantoside L, a New Tetramic Acid Glycoside with Anti-Leishmanial Activity Isolated from the Marine Sponge *Siliquariaspongia japonica*

**DOI:** 10.3390/md22040171

**Published:** 2024-04-12

**Authors:** Yasumoto Oyadomari, Yasuyuki Goto, Keisuke Suganuma, Shin-ichiro Kawazu, Leontine E. Becking, Nobuhiro Fusetani, Yoichi Nakao

**Affiliations:** 1 Department of Chemistry and Biochemistry, Graduate School of Advanced Science and Engineering, Waseda University, 3-4-1 Okubo, Shinjuku-ku, Tokyo 169-8555, Japan; yasu.10.26@ruri.waseda.jp; 2 Graduate School of Agricultural and Life Science, The University of Tokyo, Bunkyo-ku, Tokyo 113-8657, Japan; aygoto@g.ecc.u-tokyo.ac.jp; 3 National Research Center for Protozoan Diseases, Obihiro University of Agriculture and Veterinary Medicine, Inada-cho, Obihiro 080-8555, Japan; k.suganuma@obihiro.ac.jp (K.S.); skawazu@obihiro.ac.jp (S.-i.K.); 4 Aquaculture & Fisheries Group, Wageningen University & Research, P.O. Box 338, Bode 32, 6700 AH Wageningen, The Netherlands; lisa.becking@wur.nl; 5 Naturalis Biodiversity Center, Darwinweg 2, 23333 CR Leiden, The Netherlands; 6 Research Institute for Science and Engineering, Waseda University, 3-4-1 Okubo, Shinjuku-ku, Tokyo 169-8555, Japan; anobu@fish.hokudai.ac.jp

**Keywords:** aurantosides, *Siliquariaspongia japonica*, marine sponge, nuclear magnetic resonance, mass spectrometry, anti-leishmanial activity, marine natural products

## Abstract

A new tetramic acid glycoside, aurantoside L (**1**), was isolated from the sponge *Siliquariaspongia japonica* collected at Tsushima Is., Nagasaki Prefecture, Japan. The structure of aurantoside L (**1**) composed of a tetramic acid bearing a chlorinated polyene system and a trisaccharide part was elucidated using spectral analysis. Aurantoside L (**1**) showed anti-parasitic activity against *L. amazonensis* with an IC_50_ value of 0.74 µM.

## 1. Introduction

Leishmaniases are vector-borne parasitic diseases caused by several different species of *Leishmania* [1]. It is estimated that there are 12 million patients suffering from leishmaniasis with around 1 million new cases annually {www.who.int (accessed on 13 July 2023)}. Visceral leishmaniasis, caused by *L. donovani* in Asia and Africa and *L. infantum* in the Mediterranean Basin, Middle East, Central Asia, South America, and Central America, is characterized by clinical symptoms such as fever, anemia, splenomegaly, hepatomegaly, and weight loss and is fatal unless treated appropriately [2]. Cutaneous leishmaniasis is a skin manifestation that sometimes heals naturally over 3–18 months, but the ulcer can lead to serious permanent scarring, disfigurement, and stigmatization [3]. Mucocutaneous leishmaniasis leads to the partial or total destruction of the mucous membranes of the nose, mouth, and throat, and the disease can be found mostly in South America (www.who.int (accessed on 13 July 2023)). Because no effective vaccines to prevent the disease for humans are commercially available yet, the control of the disease mostly relies on chemotherapy. These leishmaniases are treated by, for example, pentavalent antimonials, liposomal amphotericin B, and milterfosine. However, drug side effects, high costs, potential teratogenicity, and the emergence of drug-resistant strains pose a serious potential threat to endemic countries where leishmaniases are prevalent [4,5].

Consequently, there have been efforts to discover new candidate compounds for chemotherapeutic use against leishmaniasis. Although target-based screening is becoming a more popular way for drug discovery, the phenotypic screening of natural compounds is still a vital choice [6]. In fact, marine bioproducts are attractive sources of anti-parasitic agents for various diseases including malaria and leishmaniasis [7]. Gracilioethers A-C were isolated from the marine sponge *Agelas gracilis* as anti-protozoan natural compounds, which had anti-malarial activity [8]. A xenicane diterpenoid, cristaxenicin A, was found in the deep-sea gorgonian *Acanthoprimnoa cristata*, showing anti-leishmanial and anti-trypanosomal activities (against *Leishmania amazonensis* and *Trypanosoma congolense*, respectively) [9].

**Figure 1:**
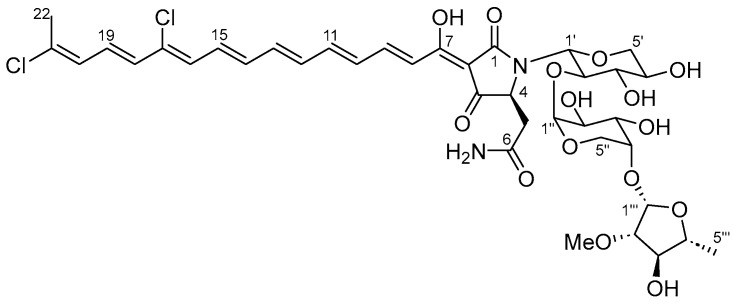
Structure of aurantoside L (**1**).

To discover potential drug leads against leishmaniasis, we focused on marine invertebrates, whose extracts are a rich source of various bioactive compounds [10]. Marine organism extracts (1565 samples) have been tested against the recombinant *L. amazonensis* doped with a green fluorescent protein (*La/egfp*). In this screening approach, a marine sponge *Siliquariaspongia japonica* extract showed strong anti-parasitic activity against *La/egfp*. From the Lithistida order of sponges, to which *S. japonica* belongs, a wide variety of compounds that are thought to be produced by the symbiotic bacteria [11] have been isolated. The marine sponge *Siliquariaspongia* sp. has been also reported several times as the source of unique and bioactive metabolites, for example, aurantosides D-E [12], rubrosides A-H [13], motualevic acids A-F [14], mirabamides A-D [15], celebesides A-C, and theopapuamides B-D [16]. Based on the result of the screening and the abundant discoveries reported so far, we considered the marine sponge *S. japonica* to be a suitable candidate for searching for substances with anti-leishmanial activity. The bioassay-guided fractionation of this sponge extract provided a new anti-leishmanial tetramic acid glycoside, aurantoside L (**1**) (Figure 1). In this paper, the isolation, structure elucidation, and biological activities of this compound are discussed.

## 2. Results

The frozen specimen of *S. japonica* (170 g, wet weight) was extracted with MeOH and CHCl_3_/CH_3_OH (1:1) repeatedly. The extracts were combined, and the concentrated extract was partitioned between H_2_O and CHCl_3_. The water-soluble layer was further extracted with *n*-C_4_H_9_OH, and the *n*-C_4_H_9_OH layer was combined with the former CHCl_3_ layer. The combined organic layer was fractionated using the Kupchan procedure [17] to yield *n*-hexane, CHCl_3,_ and 60% CH_3_OH layers. The anti-leishmanial aqueous CH_3_OH layer was subjected to octadecylsilyl (ODS) column flash chromatography to give six fractions (fr. A-F). Among them, active fr. E, eluting with 100% CH_3_OH, was subjected to reversed-phase high-performance liquid chromatography (RP-HPLC) to afford compound **1** as the active substance (12.5 mg, 7.4 × 10^−3^% yield based on the wet weight).

Compound **1** was obtained as a red amorphous solid. The electrospray ionization mass spectrum (ESIMS) (positive mode) showed clusters of ions at *m*/*z* 865, 867, and 869 [M + Na]^+^ in the ratio of 9:6:1, indicating the presence of two chlorine atoms in **1** since the natural abundance of chlorine atoms is 75% with integer mass 35 and 25% with 37. The molecular formula was established as C_38_H_48_Cl_2_N_2_O_15_ by high-resolution electrospray ionization mass spectrum (HRESIMS) (positive mode) (*m*/*z* 865.2310 [M + Na]^+^, calcd for C_38_H_48_^35^Cl_2_N_2_O_15_Na, 865.2324, Δ −1.6 mmu).

The ^1^H NMR spectrum (in CD_3_OD, at 297 K, 400 MHz) showed 12 downfield shifted signals {*δ*_H_ (6.39, d, *J* = 11.5 Hz), (6.44, d, *J* = 14.4 Hz), (6.55, d, *J* = 11.6 Hz), (6.56, m), (6.59, m), (6.61, dd, *J* = 14.5, 11.5 Hz), (6.72, dd, *J* = 16.1, 11.6 Hz), (6.75, dd, *J* = 14.4, 11.5 Hz), (6.86, dd, *J* = 14.2, 11.6 Hz), (6.89, dd, *J* = 14.5, 11.6 Hz), (7.22, d, *J* = 15.1 Hz), and (7.60, dd, *J* = 15.1, 11.5 Hz)}; 17 signals in the range of *δ*_H_ 3.21–5.09 (typical for saccharides) {*δ*_H_ (3.21, dd, *J* = 11.0, 10.8 Hz), (3.48, dd, *J* = 8.8, 8.8 Hz), (3.59, dd, *J* = 12.9, 2.3 Hz), (3.62, m), (3.67, dd, *J* = 8.1, 4.4 Hz), (3.74, dd, *J* = 12.9, 0.9 Hz), (3.76, qd, *J* = 7.1, 6.4), (3.79, dd, *J* = 9.5, 2.5 Hz), (3.80, dd, *J* = 9.5, 2.9 Hz), (3.88, dd, *J* = 10.8, 4.8 Hz), (3.90, dd, *J* = 8.1, 7.1 Hz), (3.91, m), (4.33, br), (4.52, br), (4.52, br), (5.04, br), and (5.09, d, *J* = 4.4 Hz)}; 1 vinyl methyl (*δ*_H_ 2.26, brs); 1 methoxy group (*δ*_H_ 3.35, s); and 1 doublet methyl group (*δ*_H_ 1.32, d, *J* = 6.2 Hz) （Supplementary Information）.

**Figure 2:**
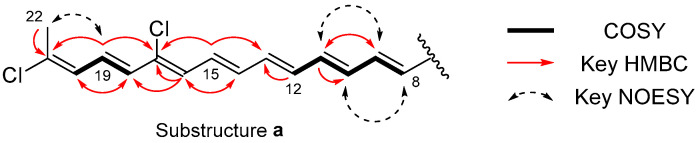
Substructure **a** deduced from COSY and key HMBC and NOESY correlations.

The interpretation of the COSY spectrum revealed the two spin systems of H-8 to H-12 {*δ*_H_ (7.22, d, *J* = 15.1 Hz)/(7.60, dd, *J* = 15.1, 11.5 Hz)/(6.61, dd, *J* = 14.5, 11.5 Hz)/(6.89, dd, *J* = 14.5, 11.6 Hz)} and H-18 to H-20 {*δ*_H_ (6.44, d, *J* = 14.4 Hz)/(6.75, dd, *J* = 14.4, 11.5 Hz)/(6.39, d, *J* = 11.5 Hz)}. The HMQC and HMBC spectra established the polyene substructure **a** (C-8 to C-22), in which quaternary carbons C-17 and C-21 were chlorinated based on the ^13^C chemical shift values (*δ*_C_ 134.7 and *δ*_C_ 136.4, respectively) [18]. The geometry of the double bonds in substructure **a,** except for *Δ*^16^, was determined as *E* based on the proton–proton coupling constants (^3^*J*_8,9_ = 15.1 Hz, ^3^*J*_10,11_ = 14.5 Hz, ^3^*J*_12,13_ = 16.1 Hz, ^3^*J*_14,15_ = 14.2 Hz, and ^3^*J*_18,19_ = 14.4 Hz), whereas that at Δ^16^ was deduced as *Z* because of the chlorine substitution at C-17. The chemical shift of C-22 (*δ*_C_ 21.8) and the NOE cross peak between H-19/H-22 indicated that the geometry at *Δ*^20^ is *E* [19,20] (Figure 2). 

COSY cross peaks in the range of *δ*_H_ 3.21–5.09 (in CD_3_OD, at 297 K, 400 MHz) showed four spin systems, H-3′/H-4′/H-5′ {*δ*_H_ (3.48, dd, *J* = 8.8, 8.8 Hz)/(3.62, m)/(3.21, dd, *J* = 11.0, 10.8 Hz)/(3.88, dd, *J* = 10.8, 4.8 Hz)}, H-1″/H-2″ {*δ*_H_ (5.04, br)/(3.80, dd, *J* = 9.5, 2.9 Hz)}, H-3″/H-4″/H-5″ {*δ*_H_ (3.79, dd, *J* = 9.5, 2.9 Hz)/(3.91, m)/(3.59, dd, *J* = 12.9, 2.3 Hz)/(3.74, dd, *J* = 12.9, 0.9 Hz)}, and H-1‴/H-2‴/H-3‴/H-4‴/Me-5 {*δ*_H_ (5.09, d, *J* = 4.4 Hz)/(3.67, dd, *J* = 8.1, 4.4 Hz)/(3.90, dd, *J* = 8.1, 7.1 Hz)/(3.76, qd, *J* = 7.1, 6.4 Hz)/(1.32, d, *J* = 6.4 Hz)}, suggesting the existence of three sugar units (sugar-I, -II and -III). HMQC and HMBC spectra (in CD_3_OD, at 297 K) indicated two of these sugars are a pyranose (sugar-II) and a 5-deoxypentofuranose (sugar-III). Sugar-II was determined to be an arabinopyranose, in which H-2″, H-3″, and H-5″ a were axial, and H-4″ and H-5″b were equatorial based on the coupling constants (^3^*J*_2″,3″_ = 9.5 Hz, ^3^*J*_3″,4″_ = 2.9 Hz, ^3^*J*_4″,5″a_ = 2.3 Hz, and ^3^*J*_4″,5″b_ = 0.9 Hz).

A methoxy group was located at C-2‴ in sugar-III by the HMBC cross peaks of H-2‴/OCH_3_ (*δ*_H_ 3.67, dd, *J* = 8.1, 4.4 Hz/*δ*_C_ 58.5) and OCH_3_/C-2‴ (*δ*_H_ 3.35, s/*δ*_C_ 87.4). The NOESY cross peaks among H-1‴/OCH_3_ (*δ*_H_ 5.09, d, *J* = 4.4/*δ*_H_ 3.35, s), OCH_3_/H-3‴ (*δ*_H_ 3.35, s/*δ*_H_ 3.90, dd, *J* = 8.1, 7.1 Hz), and H-3‴/H-5‴ (*δ*_H_ 3.90, dd, *J* = 8.1, 7.1 Hz/*δ*_H_ 1.32, d, *J* = 6.4 Hz) revealed that sugar-III was a 5-deoxy-2-*O*-methylpentofuranose.

**Figure 3:**
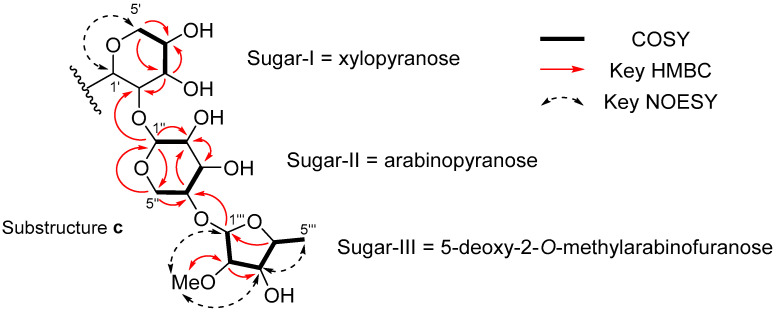
Substructure **c** deduced from COSY and key HMBC and NOESY correlations.

H-1″ and H-2″ signals in sugar-I were broadened in the ^1^H NMR spectrum at 297 K, but the distinct HMQC cross peaks among H-1′/C-1′ and H-2′/C-2′ (*δ*_H_ 4.52, br/*δ*_C_ 81.4 and *δ*_H_ 4.52, br/*δ*_C_ 86.3) were observed at a higher temperature (in CD_3_OD, at 320 K, 400 MHz). The assignment of H-1′ and H-2′ was not possible because of their overlapping signals; however, HMBC cross peaks between H-3′/C-2′ (*δ*_H_ 3.48, dd, *J* = 8.8, 8.8 Hz/*δ*_C_ 81.4) and H-1″/C-2′ (*δ*_H_ 5.04, br/*δ*_C_ 81.4) confirmed the assignment of C-2′ at this position. Along with a NOESY cross peak to H-1′/H-5′ (*δ*_H_ 5.04, br/*δ*_H_ 3.88, dd, *J* = 10.8, 4.8 Hz), coupling constants among these proton signals suggested that sugar-I was a xylopyranose. An HMBC cross peak of H-1‴/C-4″ (*δ*_H_ 5.09, d, *J* = 4.4 Hz/*δ*_C_ 79.7) indicated a sequential connection of sugars-I/II/III through *α* (1→2) and *α*,*β* (1→4), respectively (substructure **c**) (Figure 3).

**Figure 4:**
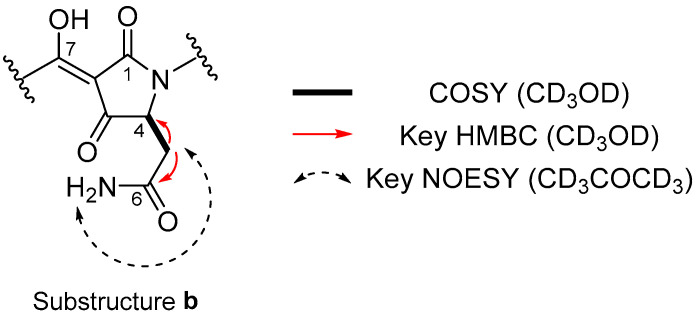
Substructure **b** deduced from COSY and key HMBC and NOESY correlations.

The remaining substructure **b** was composed of C_7_H_6_N_2_O_3_ and deduced as follows: the NMR spectra in CD_3_OD showed a CHCH_2_ spin system with a broadened H-4 proton (*δ*_H_ 4.33, br). The HMBC correlations for H-5a/C-6 (*δ*_H_ 2.67, dd, *J* = 15.4, 7.6 Hz/*δ*_C_ 174.5) and primary amide protons (1.4 H integration, *δ*_H_ 7.44, s) showing an NOE to H-5a (*δ*_H_ 2.60, dd, *J* = 14.0, 4.4 Hz) in CD_3_COCD_3_ indicated that an amide carbonyl group was connected to C-5. Although four signals for C-1 to C-4 were not observed clearly in the ^13^C NMR spectrum, the remaining constituents (one proton, five carbons, one nitrogen, and three oxygens) are typical for a tetramic acid moiety with keto-enol tautomerism [21], thus completing partial structure **b** (Figure 4).

**Figure 5:**
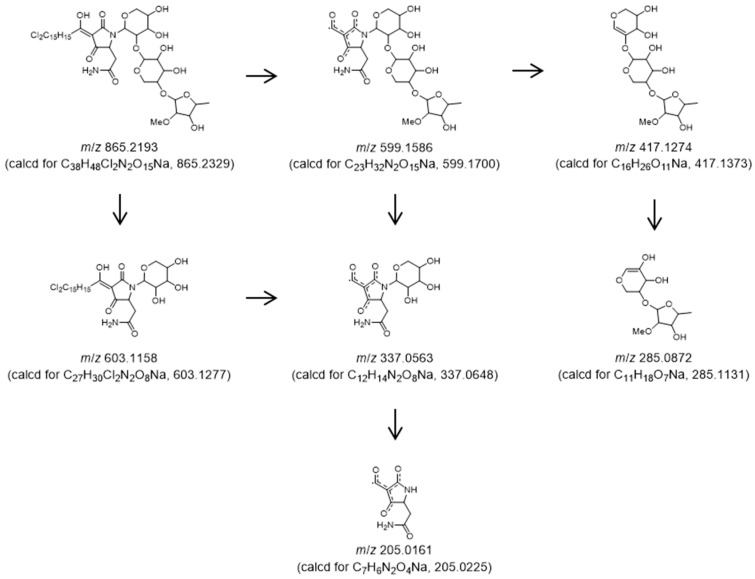
MS/MS fragmentations of aurantoside L (**1**).

The whole planar structure of **1** was constructed using HMBC and MS/MS data analysis. HMBC correlations for H-8/C-7 (*δ*_H_ 7.22, d, *J* = 15.1 Hz/*δ*_C_ 175.2) and H-9/C-7 (*δ*_H_ 7.60, dd, *J* = 15.1, 11.5 Hz/*δ*_C_ 175.2) observed in CD_3_OD at 320 K indicated that partial structures **a** and **b** were connected between C-8 and C-7. MS/MS (positive ion mode) fragmentation analysis resolved the connection of partial structures **a**, **b**, and **c** and the sequence of the trisaccharide. The ion giving *m*/*z* 865.2193 (composed of C_38_H_48_Cl_2_N_2_O_15_Na) was chosen as the precursor ion for the experiment. The intensity of the sodium-cationized ion peak at *m*/*z* 599.1586 (calcd for C_23_H_32_N_2_O_15_Na, 599.1700) was the strongest among the fragment ion peaks observed, suggesting the conjugated system in the tetramic acid moiety was formed by the desorption of substructure **a** and stabilized in MS/MS fragmentation. The second strongest fragment ion peak appeared as *m*/*z* 337.0563 (calcd for C_12_H_14_N_2_O_8_Na, 337.0648), which was thought to be composed of the tetramic acid moiety (*m*/*z* 205.0161) and the xylopyranose (sugar-I). Besides these peaks, fragment ion peaks at *m*/*z* 603.1158 (intermediate ion peaks from *m*/*z* 865.2193 to 337.0563) and *m*/*z* 417.1274 to 285.0872 corresponding to the sugar sequence also supported the structure deduced by NMR experiments (see Figure 5). These experiments confirmed the planar structure of compound **1** as a new tetramic acid glycoside, aurantoside L (**1**) (Figure 5).

According to the literature, aurantosides A-F and rubrosides A-H are all derived from L-aspartic acid (detected via the GC analysis of the acid hydrolysate of the Lemieux oxidation product) and carry D-saccharides [12,13,21,22,23,24,25]. This structural information indicates that a common biosynthetic pathway produces these polyene tetramic acid glycosides. Based on biogenetic reasoning and comparing spectroscopic data with those of analogs, the absolute configurations at C-4 and each saccharide were presumed to be identical to that of analogs. Therefore, the absolute configuration of aurantoside L (**1**) was tentatively assigned as 4*S* and the saccharides as D-forms.

Aurantoside L (**1**) exhibited anti-leishmanial activity against *La/egfp* with an IC_50_ value of 0.74 µM, while it showed modest cytotoxicity against HeLa cells and P388 cells with IC_50_ values of 2.4 and 1.1 µM, respectively. In contrast, aurantoside L (**1**) was inactive at 3.0 µM against *Trypanosoma congolense*, indicating selective anti-parasitic activity within the same family of Trypanosomatidae.

## 3. Materials and Methods

### 3.1. General Experimental Procedures

NMR spectra were recorded on an Avance (400 MHz) spectrometer (Bruker Corporation, Billerica, MA, USA). ^1^H and ^13^C NMR chemical shifts were referenced to the CD_3_OD solvent peaks *δ*_H_ 3.31 and *δ*_C_ 49.15 (Wako, Osaka, Japan). HRESI-MS spectra were measured on an Exactive Plus (Thermo Fisher Scientific Inc., Waltham, MA, USA). ESIMS/MS spectra were measured on a TripleTOF 4600 (AB Sciex Pte. Ltd., Tokyo, Japan) in the positive mode. Optical rotation was determined on a P-2200 polarimeter (JASCO Corporation, Tokyo, Japan) in CH_3_OH. UV spectra were recorded using a V-630 spectrophotometer (JASCO). IR spectra were measured on a Nicolet6700 spectrometer (Thermo Fisher Scientific Inc.).

### 3.2. Biological Material

*S. japonica* was collected by hand using SCUBA (13 m depth, on rocky shores), Tsushima Is., Nagasaki Prefecture, Japan (N 34°15′30″, E 129°19′50″) in June 2008. The sample was immediately frozen and stored at −25 °C until extraction.

### 3.3. Isolation

The frozen *S. japonica* (170 g, wet weight) was extracted with CH_3_OH and then with CHCl_3_/CH_3_OH (1:1). The concentrated extract was suspended in H_2_O and extracted with CHCl_3_ and *n*-C_4_H_9_OH. The CHCl_3_ and *n*-C_4_H_9_OH layers were combined and subjected to the Kupchan procedure to yield *n*-hexane, CHCl_3,_ and aqueous CH_3_OH layers. The aqueous CH_3_OH layer was concentrated to dryness and separated by ODS flash chromatography (H_2_O/CH_3_OH = 100/0, 80/20, 50/50, 30/70, and 0/100, and CHCl_3_/CH_3_OH/H_2_O = 60/40/10) to give six fractions (fr. A–F). Among them, active fr. E was purified with ODS HPLC (COSMOSIL 5C_18_ AR-II 20 × 250 mm, 50% CH_3_CN + 0.05% TFA, 8 mL/min) to afford 12.5 mg of aurantoside L (**1**, 7.4 × 10^−3^% yield based on the wet weight).

*Aurantoside L* (**1**): red amorphous solid; [α]_D_^23^ −36° (c 0.001, CH_3_OH); UV (H_2_O) *λ*_max_ (log ε) 427 (3.79), 250 (3.56) nm; UV (0.01 N HCl) *λ*_max_ (log ε) 428 (3.77), 329 (3.65) nm; UV (0.01 N NaOH) *λ*_max_ (log ε) 435 (4.90), 251 (4.24) nm; IR (KBr film) *ν*_max_ 3350, 1613, 1576, 1530, 1404, 1073, and 1005 cm^−1^; HRESIMS *m*/*z* 865.2310 [M + Na]^+^ (calcd for C_38_H_48_^35^Cl_2_N_2_O_15_Na, 865.2324. *Δ* −1.6 mmu); ^1^H and ^13^C NMR data; see Table 1.

### 3.4. Anti-Leishmanial Assay

*La/egfp* promastigotes (1 × 10^5^ cells) were cultured for 72 h in 199 medium (NISSUI Pharmaceutical, Tokyo, Japan) in 96-well plates with various concentrations of marine invertebrate extracts, as previously reported [26]. Fluorescence was measured with excitation at 485 nm and emission at 538 nm.

### 3.5. Anti-Trypanosomal Assay

The procyclic form of the parasite (2 × 10^5^ cells per well) *Trypanosoma congolense* IL 3000 was cultured for 48 h in TVM-1 medium [27] in 96-well plates with various concentrations of aurantoside L (**1**). Ten microliters of TetraColor ONE (Seikagaku Biobusiness, Tokyo, Japan) was added to each well. After 4 h, the absorbance of the samples was read at 450 nm using a microplate reader.

### 3.6. Cell Culture

HeLa human cervical cancer cells were cultured at 37 °C under an atmosphere of 5% CO_2_ in Dulbecco’s modified Eagle’s medium (DMEM, Low Glucose, Wako, Osaka, Japan), containing 10% fetal bovine serum (FBS, Biowest, Nuaillé, France), 2 µg/mL of gentamicin, and 10 µg/mL of antibiotic-antimycotic. P388 murine leukemia cells were propagated and maintained at 37 °C under an atmosphere of 5% CO_2_ in Roswell Park Memorial Institute medium (RPMI, Wako), containing HRDS solution (2,2′-dithiobisethanol) and kanamycin sulfate.

### 3.7. Cytotoxic Test

HeLa human cervical cancer cells in DMEM (cell concentration, 10,000 cells/mL, 200 µL) were added to each well of the microplates (96-well microplates, Costar, Washington, DC, USA) and kept in the incubator at 37 °C under an atmosphere of 5% CO_2_ for 24 h. The sample solution (2 µL in MeOH or DMSO) at 1 mg/mL was added to each well with the medium. As the positive control, 2 µL of 1 mg/mL adriamycin was added to a well of each microplate. One-fourth of this medium (ca. 50 µL) with a sample was transferred to a second well with medium (200 µL) to give a 1/5 dilution of the sample concentration. Two or six additional dilution steps gave four or eight sample concentrations. The prepared sample solutions (200 µL) were transferred to wells seeded with HeLa cells and then cultured at 37 °C under an atmosphere of CO_2_ for 72 h. Cytotoxic tests against P388 murine leukemia cells were carried out in the same manner except for the medium (RPM1 medium, as described in Cell Culture). After 72 h of cultivation, 50 µL of 3-(4,5-dimethyl-2-thiazoyl)-2,5-diphenyl-2H tetrazolium bromide (MTT) saline solution (1 mg/mL) was added to each well and the sample further incubated at 37 °C under an atmosphere of 5% CO_2_. After 4 h, the medium was removed via aspiration, and 150 µL of CH_3_COCH_3_ was added to each well to lyse the cells. The concentration of the reduced MTT was quantified by measuring the absorbance at 650 nm to estimate IC_50_ values.

## 4. Conclusions

Bioassay-guided isolation for anti-leishmanial activity afforded a new tetramic acid glycoside, aurantoside L (**1**), from the marine sponge *S. japonica*. The structure was elucidated using NMR and MS analyses. By combining high-temperature measurements and 2D NMR in different deuterated NMR solvents, the broadened ^1^H signals and the unobserved ^13^C signals that are measured by 1D NMR in CD_3_OD at room temperature were successfully assigned. Since the MS/MS experiment gives remarkably characteristic fragment ions for tetramic acid glycosides with polyene side chains, it was found to be useful for the structural analysis of compounds containing a tetramic acid moiety such as aurantoside analogs. Cytotoxicity against leukemia cells and antifungal activity have been reported for aurantoside analogs so far. There is a report that the number and structure of saccharide moiety are related to the strength and selectivity of bioactivity [21]. Notably, this is the first report of a tetramic acid glycoside exhibiting anti-leishmanial activity [28]. The unique structure and strong activity of aurantoside L (**1**) indicate a novel mechanism of action, which may lead to the development of a new treatment of leishmaniases.

## Figures and Tables

**Table 1:** NMR data of aurantoside L (**1**) in CD_3_OD at 297 K.

Position	*δ*_C_	*δ*_H_ Mult. (J in Hz)	COSY	HMBC
1				
2				
3				
4		4.33 br	5a, 5b	
5a	38.4	2.67 dd (15.4, 7.6)	4, 5b	6
5b		2.81 dd (15.4, 3.6)	4, 5a	6
6	174.5			
7	175.2			
8	122.3	7.22 d (15.1)	9	7, 10
9	146.5	7.60 dd (15.1, 11.5)	8, 10	7, 11
10	133.6	6.61 dd (14.5, 11.5)	9, 11	12
11	145.3	6.89 dd (14.5, 11.6)	10, 12	9
12	135.9	6.56 m	11	13
13	140.5	6.72 dd (16.1, 11.6)		11, 15
14	137.7	6.59 m		16
15	132.9	6.86 dd (14.2, 11.6)		13, 17
16	131.3	6.55 d (11.6)		14, 17, 18
17	134.7			
18	131.8	6.44 d (14.4)	19	16, 17, 20
19	128.0	6.75 dd (14.4, 11.5)	18, 20	17, 21
20	129.0	6.39 d (11.5)	19, 22	18, 21, 22
21	136.4			
22	21.8	2.26 brs	20	20, 21
1′	86.3	4.52 br		
2′	81.4	4.52 br		
3′	79.4	3.48 dd (8.8, 8.8)	4′	2′, 4′
4′	70.6	3.62 m	3′, 5′a, 5′b	
5′a	69.3	3.21 dd (11.0, 10.8)	4′, 5′b	3′, 4′
5′b		3.88 dd (10.8, 4.8)	4′, 5′a	4′
1″	104.0	5.04 br	2″	2′, 2″, 5″
2″	71.7	3.80 dd (9.5, 2.9)	1″	3″
3″	70.9	3.79 dd (9.5, 2.9)	4″	2″
4″	76.1	3.91 m	3″, 5″a, 5″b	2″
5″a	61.6	3.59 dd (12.9, 2.3)	4″, 5″b	1″, 3″, 4″
5″b		3.74 dd (12.9, 0.9)	4″, 5″a	
1‴	98.9	5.09 d (4.4)	2‴	4″, 2‴, 3‴
2‴	87.4	3.67 dd (8.1, 4.4)	1‴, 3‴	1‴, 3‴, OMe
3‴	79.9	3.90 dd (8.1, 7.1)	2‴, 4‴	1‴, 2‴, 4‴, 5‴
4‴	79.7	3.76 qd (7.1, 6.4)	3‴, 5‴	1‴, 3‴
5‴	21.0	1.32 d (6.4)	4‴	4‴
OCH_3_	58.5	3.35 s		2‴

## Data Availability

Data from the present study are available in the article and Supplementary Materials.

## Supplementary Materials

The following supporting information can be downloaded at https://www.mdpi.com/article/10.3390/md22040171/s1: Figures S1–S19: NMR and MS spectra of compound **1**.

## Sample Availability

Samples of the compounds are available from the authors.
