# Supplementary material for: Aurantoside L, a New Tetramic Acid Glycoside with Anti-Leishmanial Activity Isolated from the Marine Sponge Siliquariaspongia japonica
Source: Mar Drugs. 2024 Apr 12;22(4):171. doi: 10.3390/md22040171 (PMC11050911; doi:10.3390/md22040171)
Supplement: Supplementary file 1 [file marinedrugs-22-00171-s001.zip › marinedrugs-2939756-supplementary.pdf]

Supporting Information

Aurantoside L, a New Tetramic Acid Glycoside with
Anti-Leishmanial Activity Isolated from the Marine Sponge *Siliquariaspongia japonica*

Yasumoto Oyadomari [1], Yasuyuki Goto [2], Keisuke Suganuma [3], Shin-ichiro Kawazu [3], Leontine E. Becking [4,5],
Nobuhiro Fusetani [6] and Yoichi Nakao [1,6,*]

[1] Department of Chemistry and Biochemistry, Graduate School of Advanced Science and Engineering, Waseda University, 3-4-1 Okubo, Shinjuku-ku, Tokyo 169-8555, Japan; yasu.10.26@ruri.waseda.jp
[2] Graduate School of Agricultural and Life Science, The University of Tokyo, Bunkyo-ku, Tokyo 113-8657, Japan; aygoto@g.ecc.u-tokyo.ac.jp
[3] National Research Center for Protozoan Diseases, Obihiro University of Agriculture and Veterinary Medicine, Inada-cho, Obihiro, Hokkaido 080-8555, Japan; k.suganuma@obihiro.ac.jp (K.S.); skawazu@obihiro.ac.jp (S.-i.K.)
[4] Aquaculture & Fisheries Group, Wageningen University & Research, P.O. Box 338, Bode 32, 6700 AH Wageningen, The Netherlands; lisa.becking@wur.nl
[5] Naturalis Biodiversity Center, Darwinweg 2, 23333 CR Leiden, The Netherlands
[6] Research Institute for Science and Engineering, Waseda University, 3-4-1 Okubo, Shinjuku-ku, Tokyo 169-8555, Japan; anobu@fish.hokudai.ac.jp
* Correspondence: ayocha@waseda.jp; Tel.: +813-5286-3100

Table of contents

Figure S1. 1D ^{1}H NMR spectrum of aurantoside L (**1**) (CD_3OD, 297 K).

Figure S2. 1D ^{13}C NMR spectrum of aurantoside L (**1**) (CD_3OD, 297 K).

Figure S3. DEPT-135 spectrum of aurantoside L (**1**) (CD_3OD, 297 K).

Figure S4. 2D ^{1}H-^{1}H COSY of aurantoside L (**1**) (CD_3OD, 297 K).

Figure S5. 2D ^{1}H-^{1}H COSY of aurantoside L (**1**) in the range from 6.0 to 8.0 ppm (CD_3OD, 297 K).

Figure S6. 2D ^{1}H-^{13}C HMQC HMQC spectrum of aurantoside L (**1**) (CD_3OD, 297 K).

Figure S7. 2D ^{1}H-^{13}C HMQC HMBC spectrum of aurantoside L (**1**) (CD_3OD, 297 K).

Figure S8. 2D ^{1}H-^{1}H NOESY spectrum of aurantoside L (**1**) (CD_3OD, 297 K).

Figure S9. 1D ^{1}H NMR spectrum of aurantoside L (**1**) (CD_3OD, 320 K).

Figure S10. 1D ^{13}C NMR spectrum of aurantoside L (**1**) (CD_3OD, 320 K).

Figure S11. DEPT-135 spectrum of aurantoside L (**1**) (CD_3OD, 320 K).

Figure S12. 2D ^{1}H-^{1}H COSY spectrum of aurantoside L (**1**) (CD_3OD, 320 K).

Figure S13. 2D ^{1}H-^{13}C HMQC HMQC spectrum of aurantoside L (**1**) (CD_3OD, 320 K).

Figure S14. 2D ^{1}H-^{13}C HMQC HMBC spectrum of aurantoside L (**1**) (CD_3OD, 320 K).

Figure S15. 2D ^{1}H-^{1}H NOESY spectrum of aurantoside L (**1**) (CD_3OD, 320 K).

Figure S16. 1D ^{1}H NMR spectrum of aurantoside L (**1**) (CD_3COCD_3, 297 K).

Figure S17. 2D ^{1}H-^{1}H NOESY spectrum of aurantoside L (**1**) (CD_3COCD_3, 297 K).

Figure S18. ESIMS of aurantoside L (**1**) (positive mode).

Figure S19. ESIMS/MS of aurantoside L (**1**) (positive mode, collision energy = 40 eV, precursor ion = *m*/*z* 865.2).

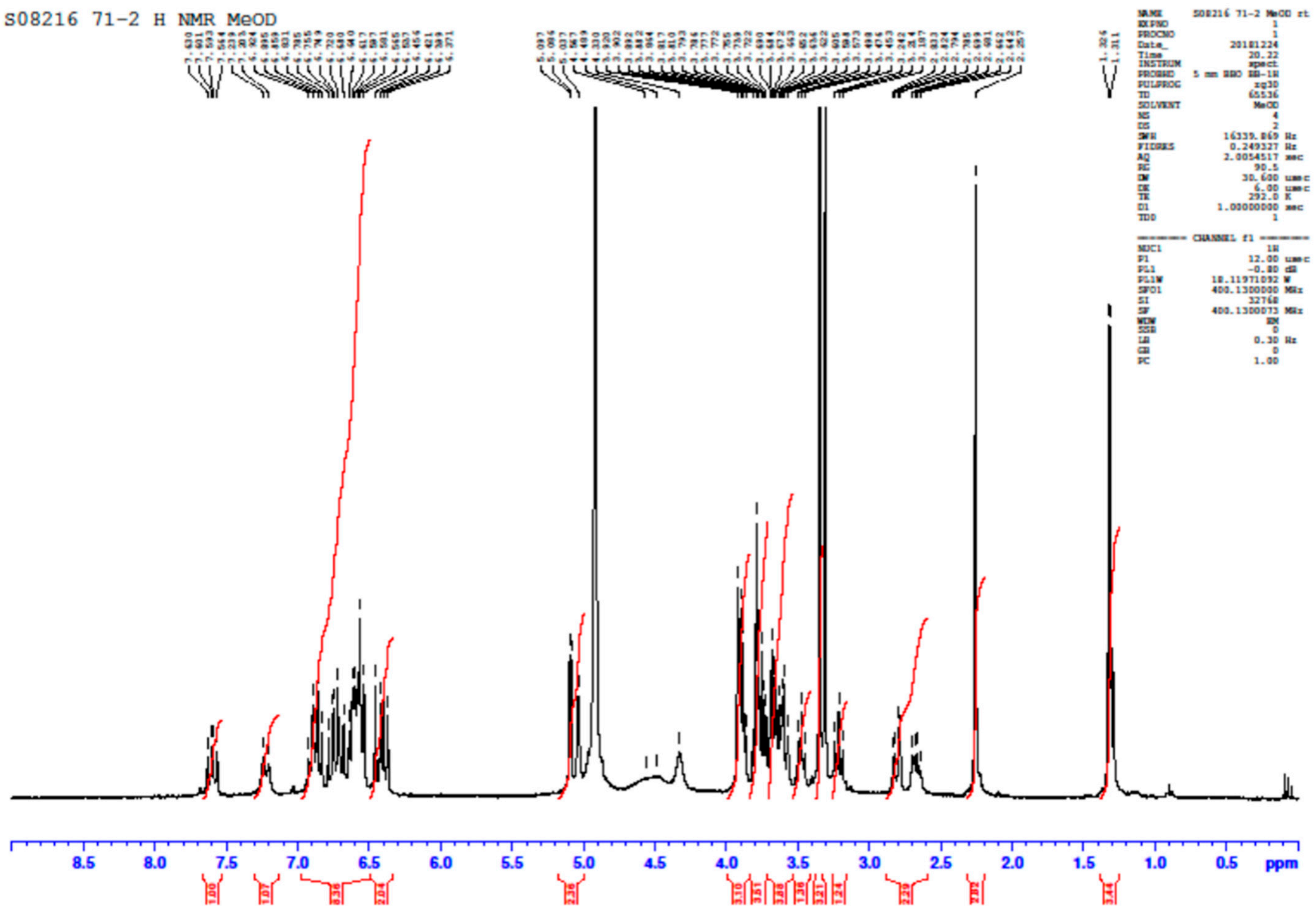

Figure S1. 1D ^{1}H NMR spectrum of aurantoside L (**1**) (CD_3OD, 297 K).

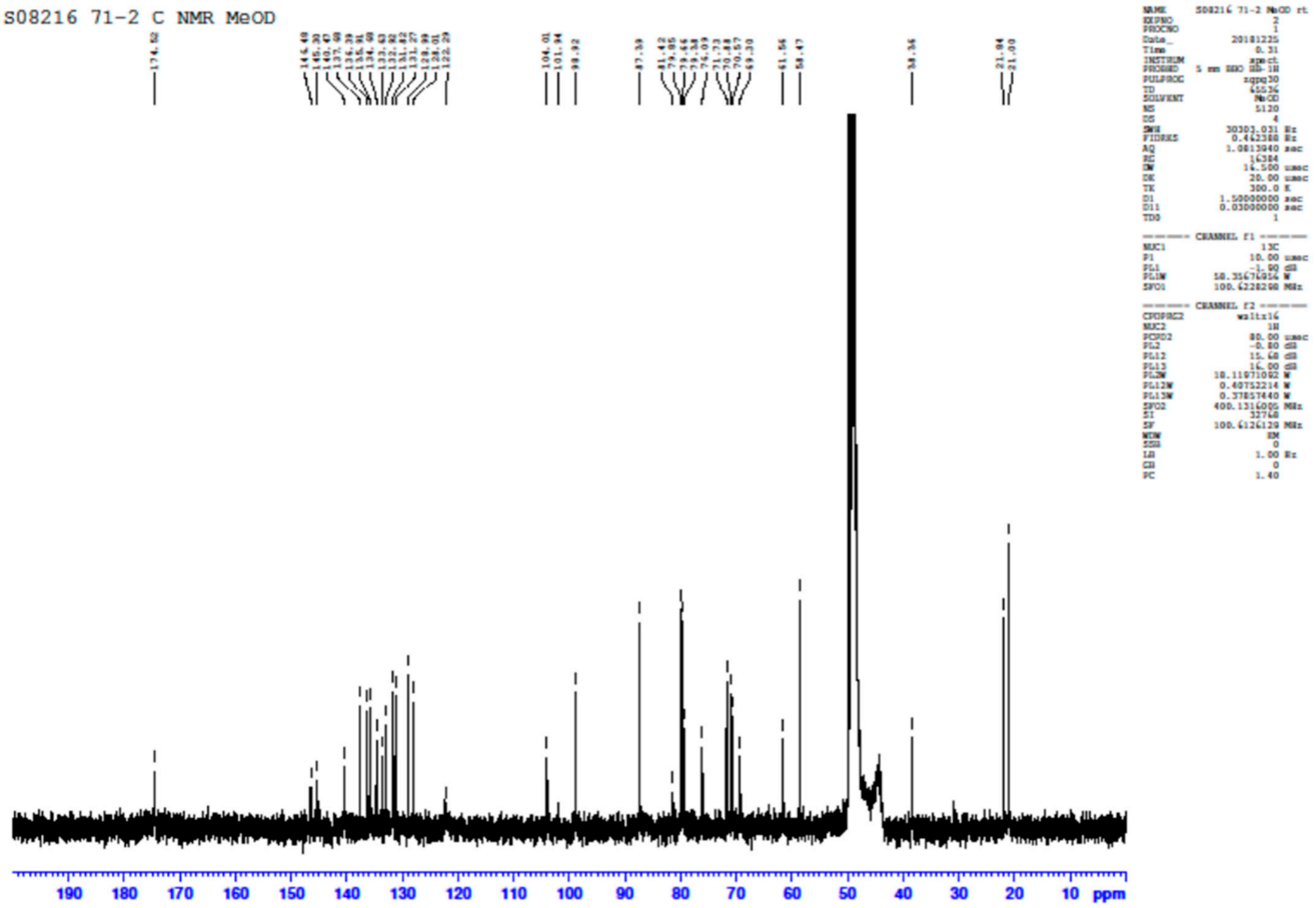

Figure S2. 1D ^{13}C NMR spectrum of aurantoside L (**1**) (CD_3OD, 297 K).

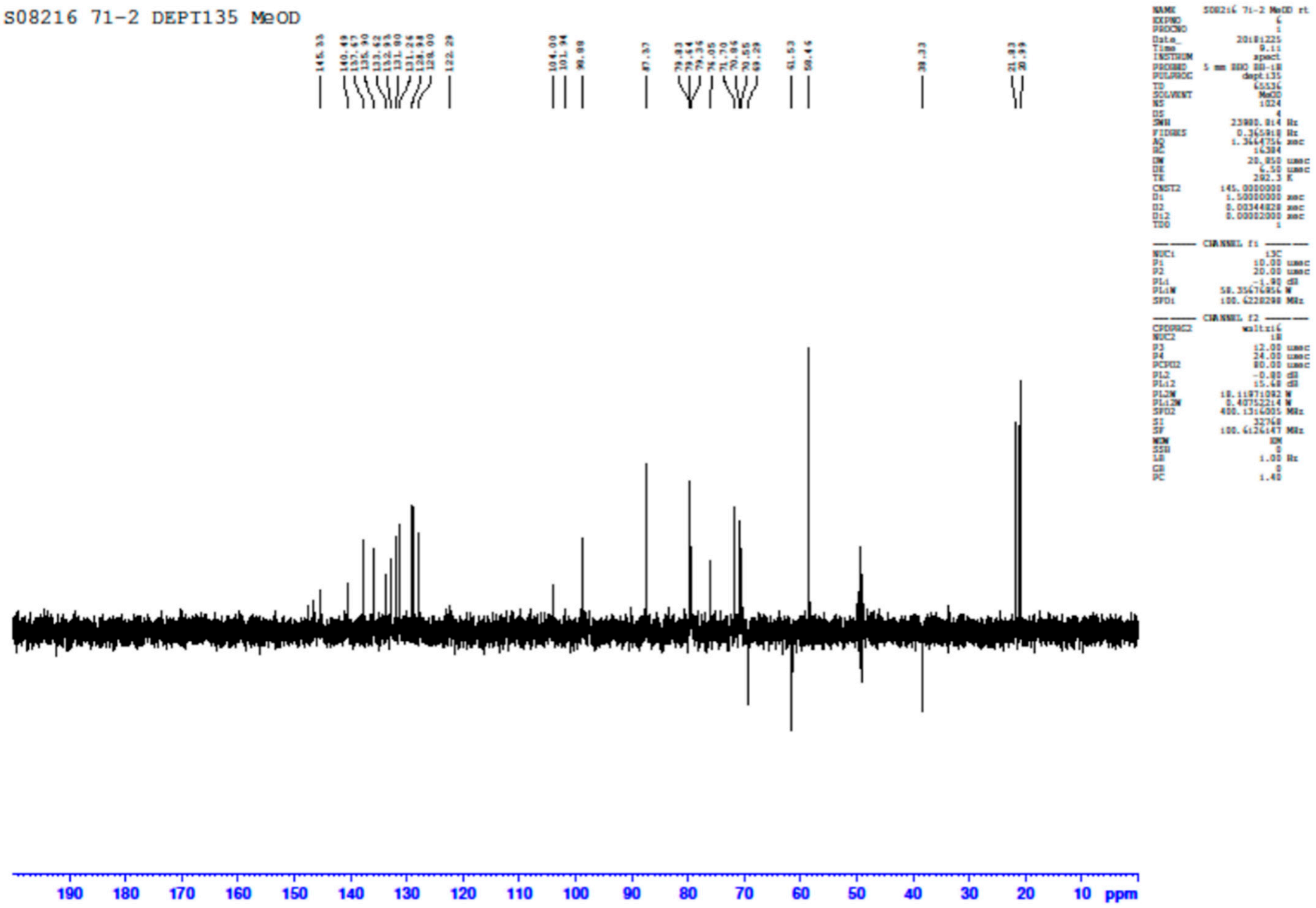

Figure S3. DEPT-135 spectrum of aurantoside L (**1**) (CD_3OD, 297 K).

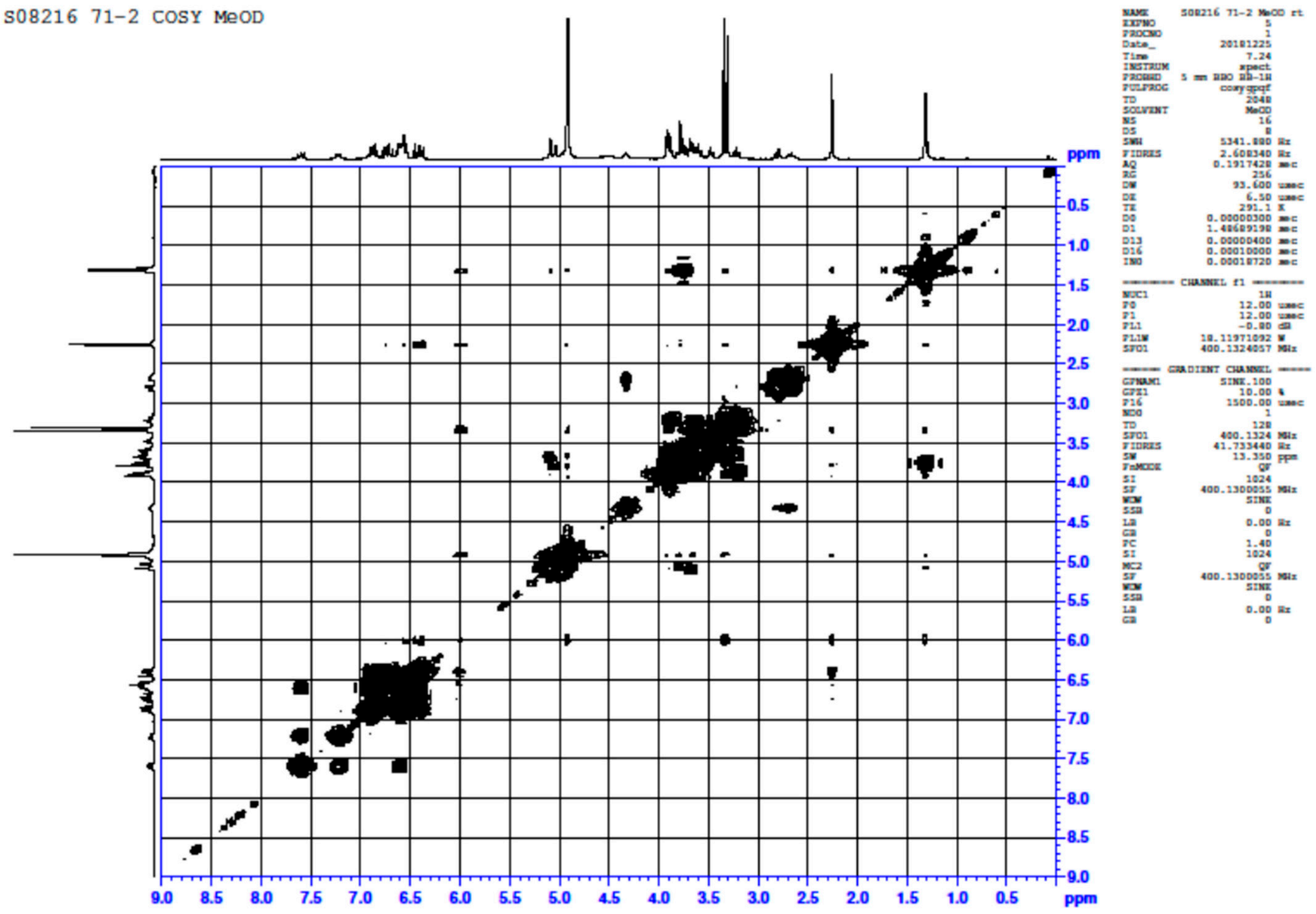

Figure S4. 2D ^1H-^1H COSY spectrum of aurantoside L (**1**) (CD_3OD, 297 K).

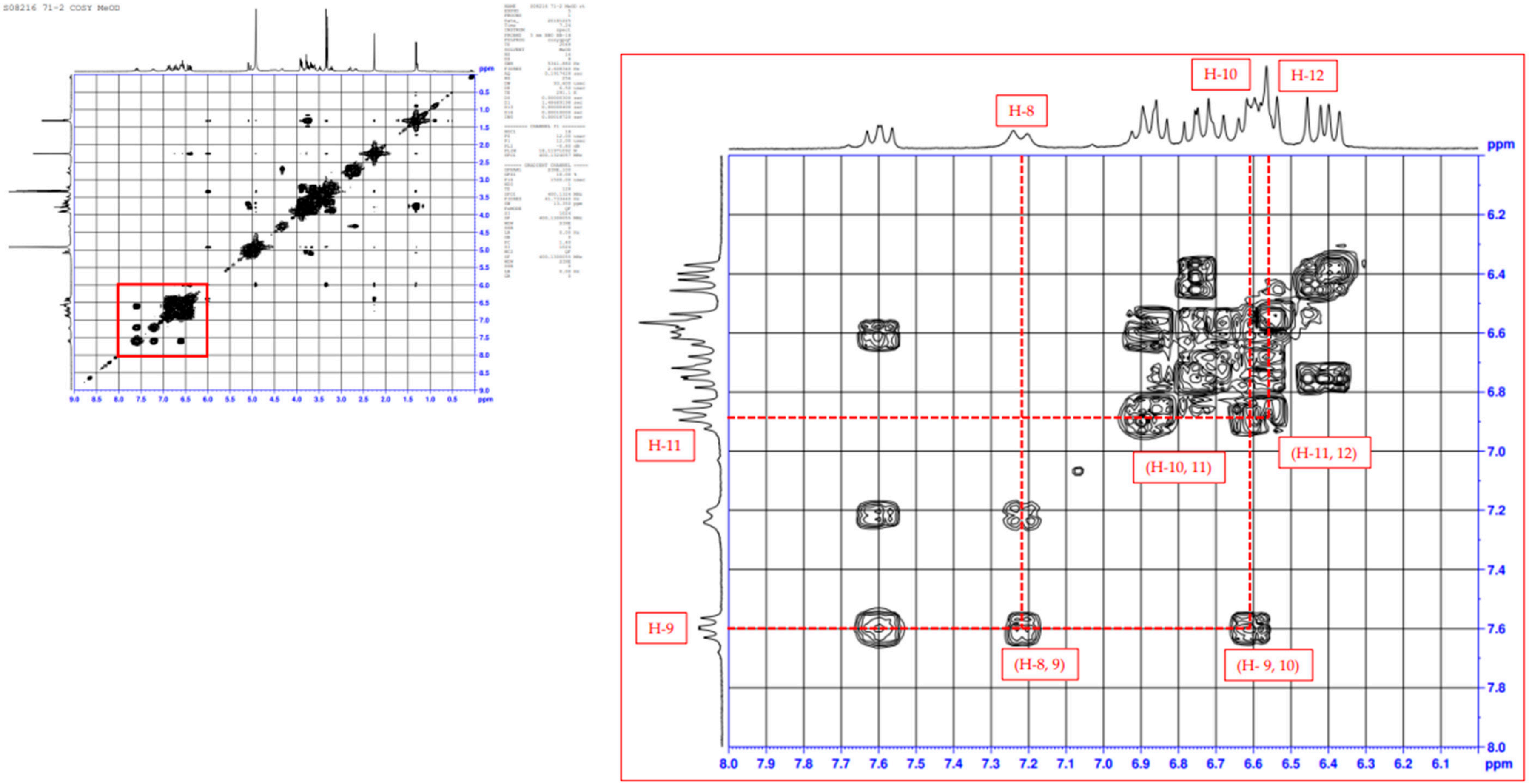

Figure S5. 2D ^{1}H-^{1}H COSY spectrum of aurantoside L (**1**) in the range from 6.0 to 8.0 ppm (CD_3OD, 297 K).

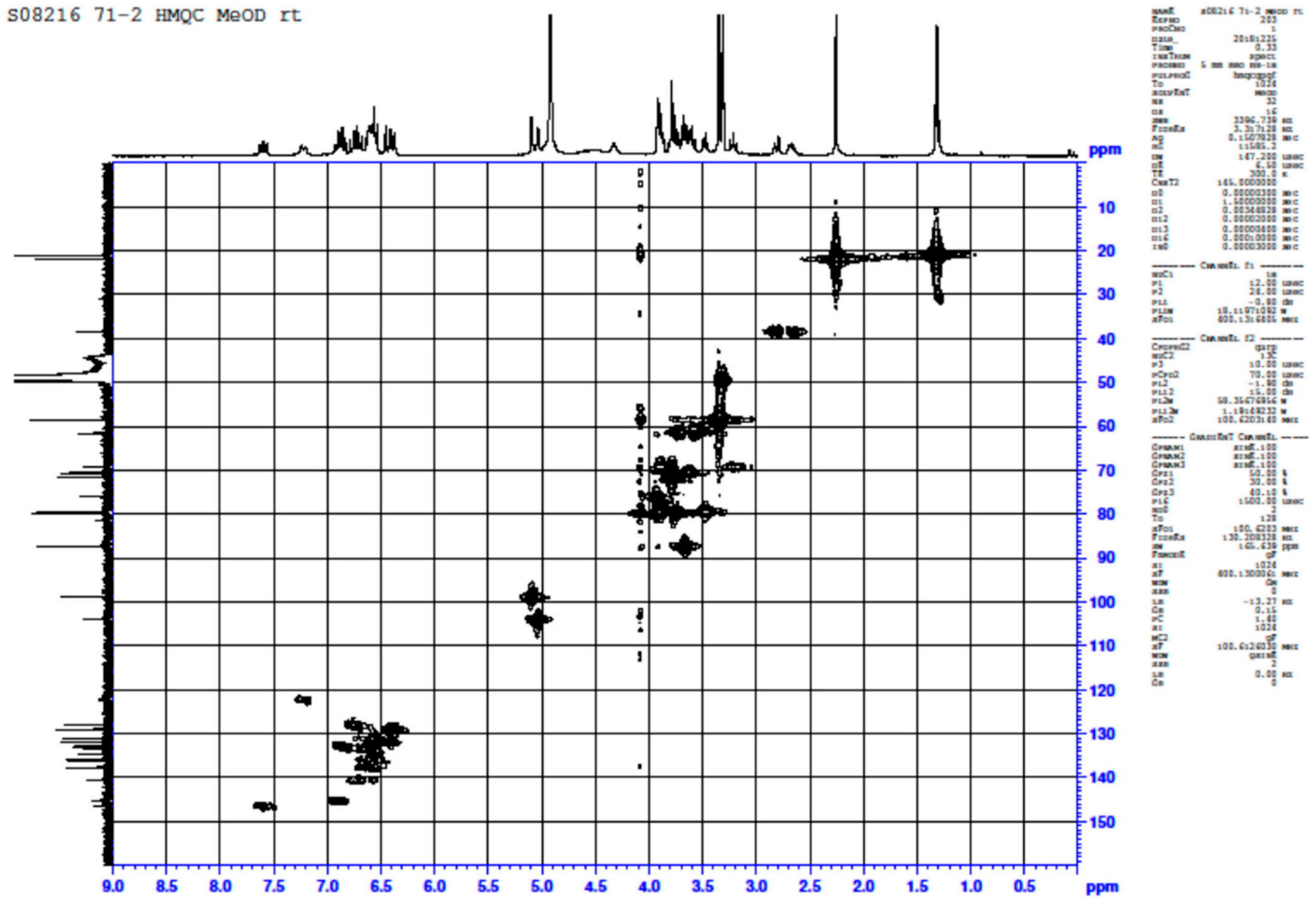

Figure S6. 2D ^{1}H-^{13}C HMQC spectrum of aurantoside L (**1**) (CD_3OD, 297 K).

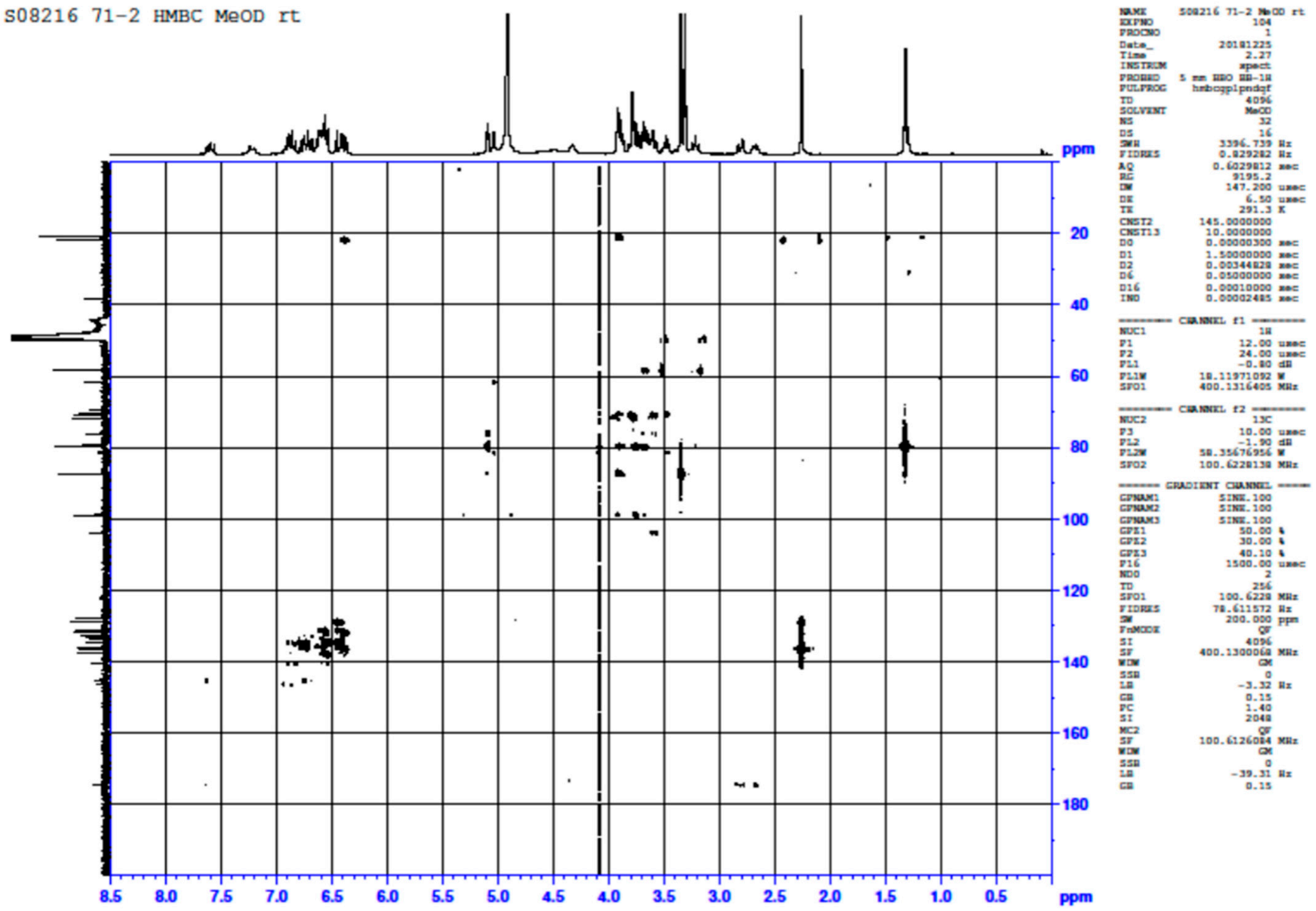

Figure S7. 2D ^{1}H-^{13}C HMBC spectrum of aurantoside L (**1**) (CD_3OD, 297 K).

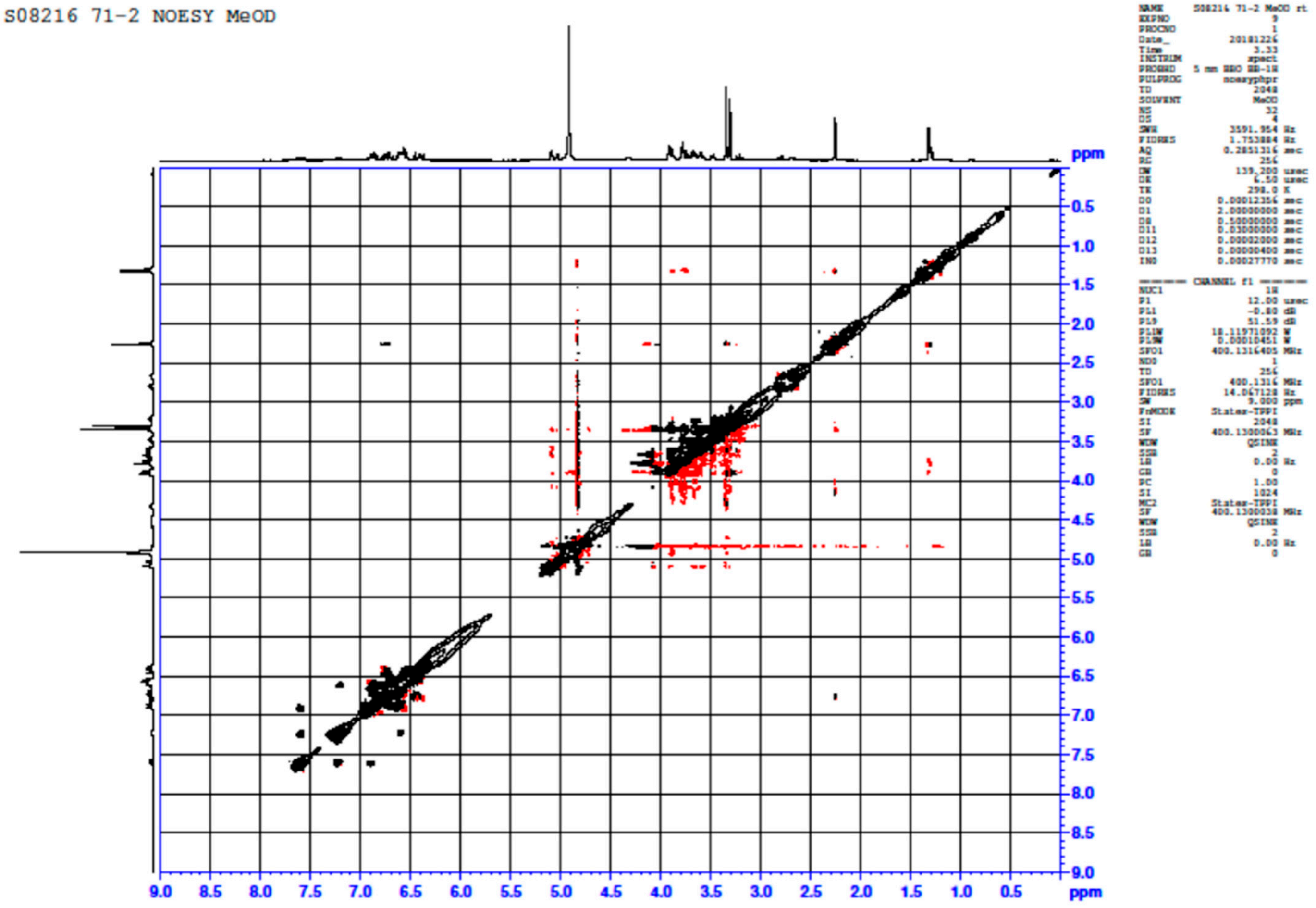

Figure S8. NOESY spectrum of aurantoside L (**1**) (CD_3OD, 297 K).

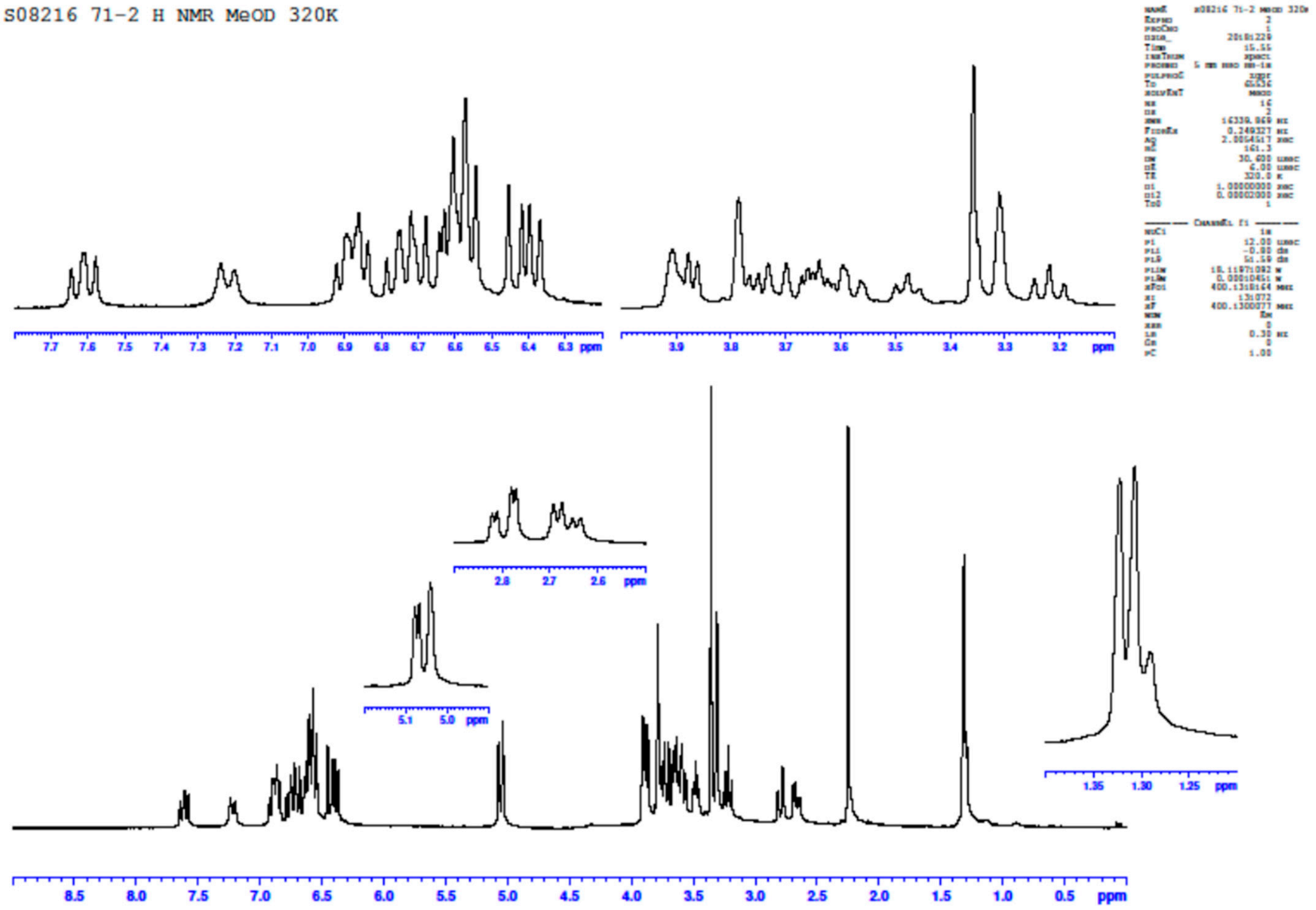

Figure S9. 1D ^{1}H NMR spectrum of aurantoside L (**1**) (CD_3OD, 320 K).

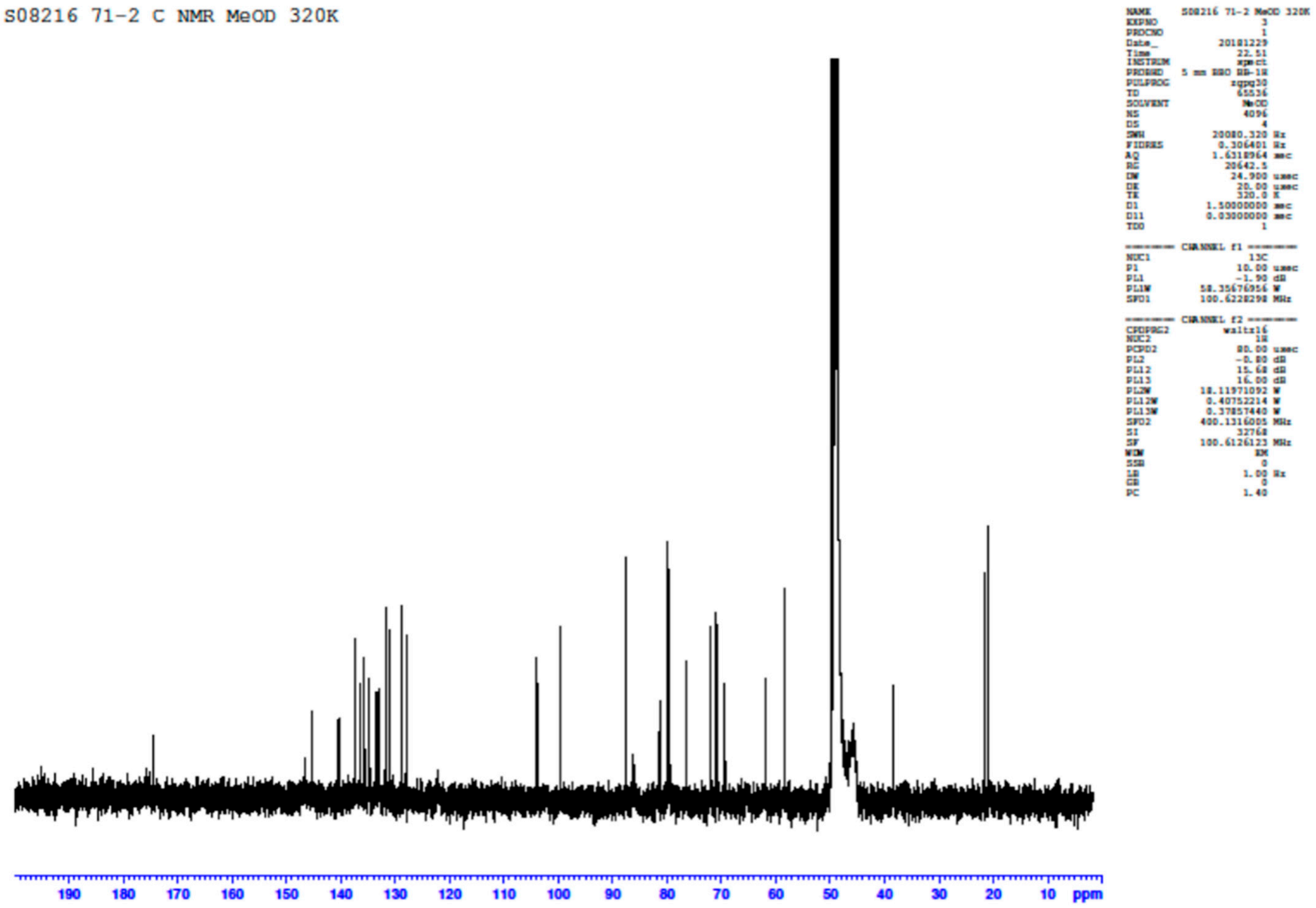

Figure S10. 1D ^{13}C NMR spectrum of aurantoside L (**1**) (CD_3OD, 320 K).

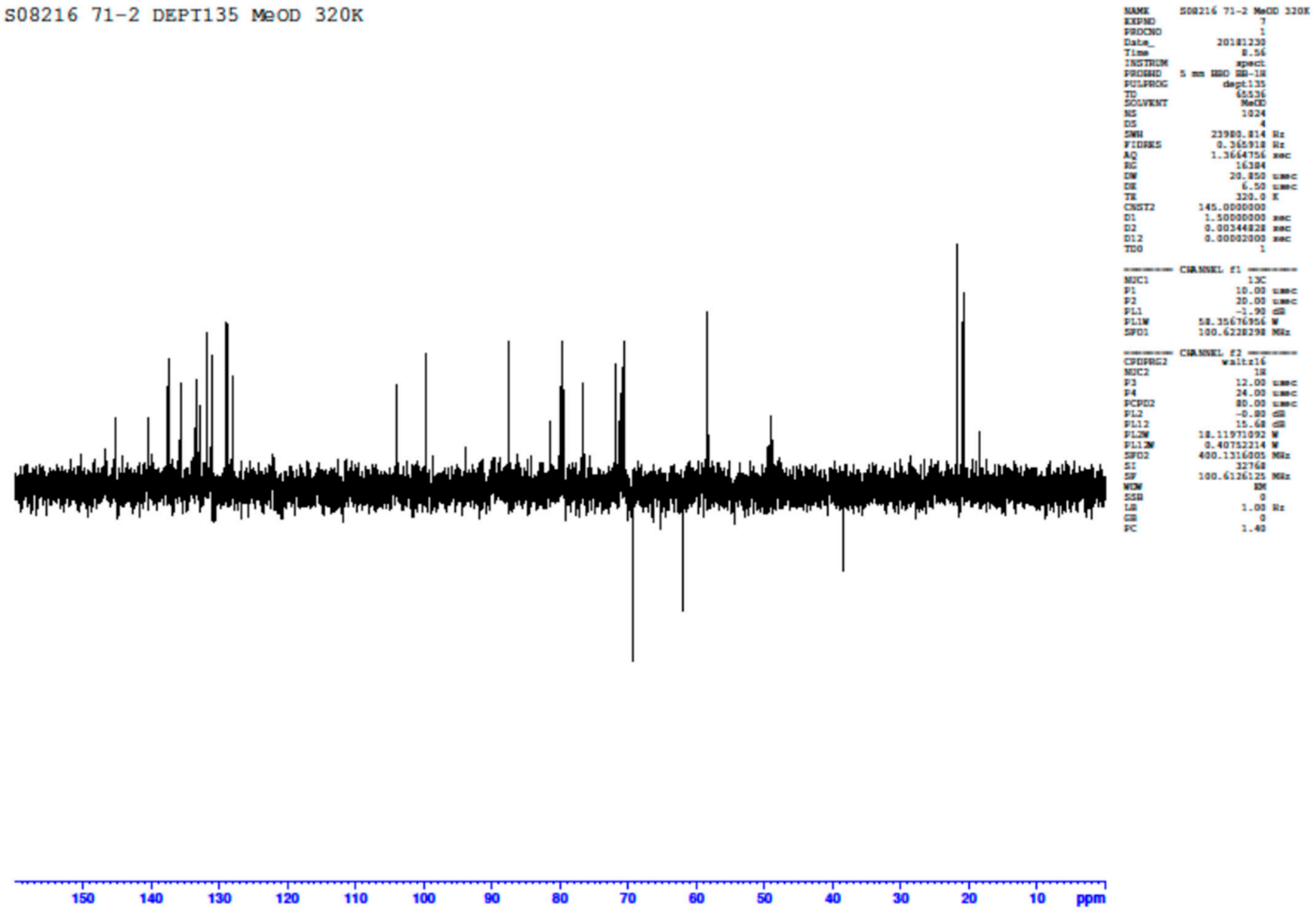

Figure S11. DEPT-135 spectrum of aurantoside L (**1**) (CD_3OD, 320 K).

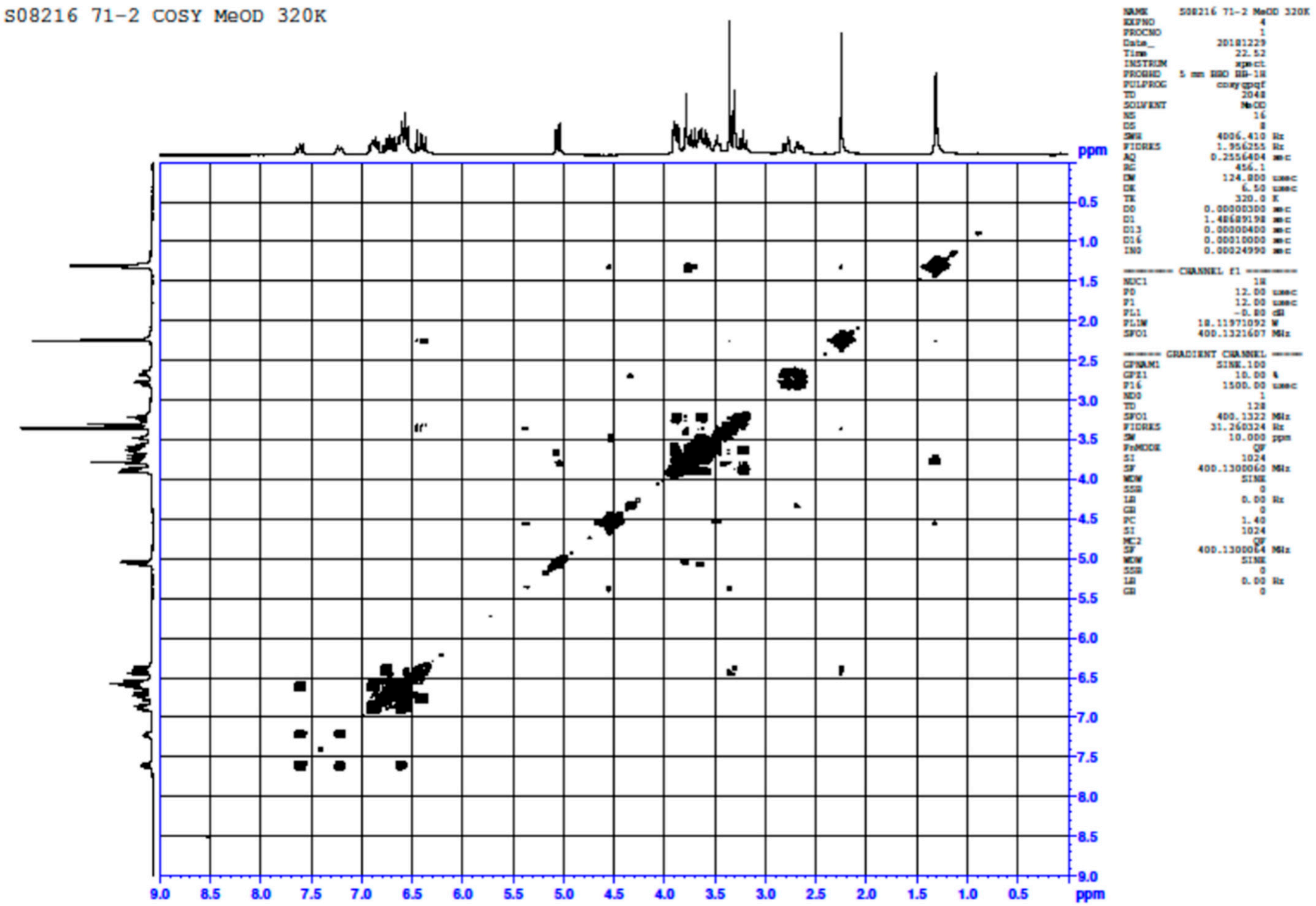

Figure S12. 2D ^{1}H-^{1}H COSY spectrum of aurantoside L (**1**) (CD_3OD, 320 K).

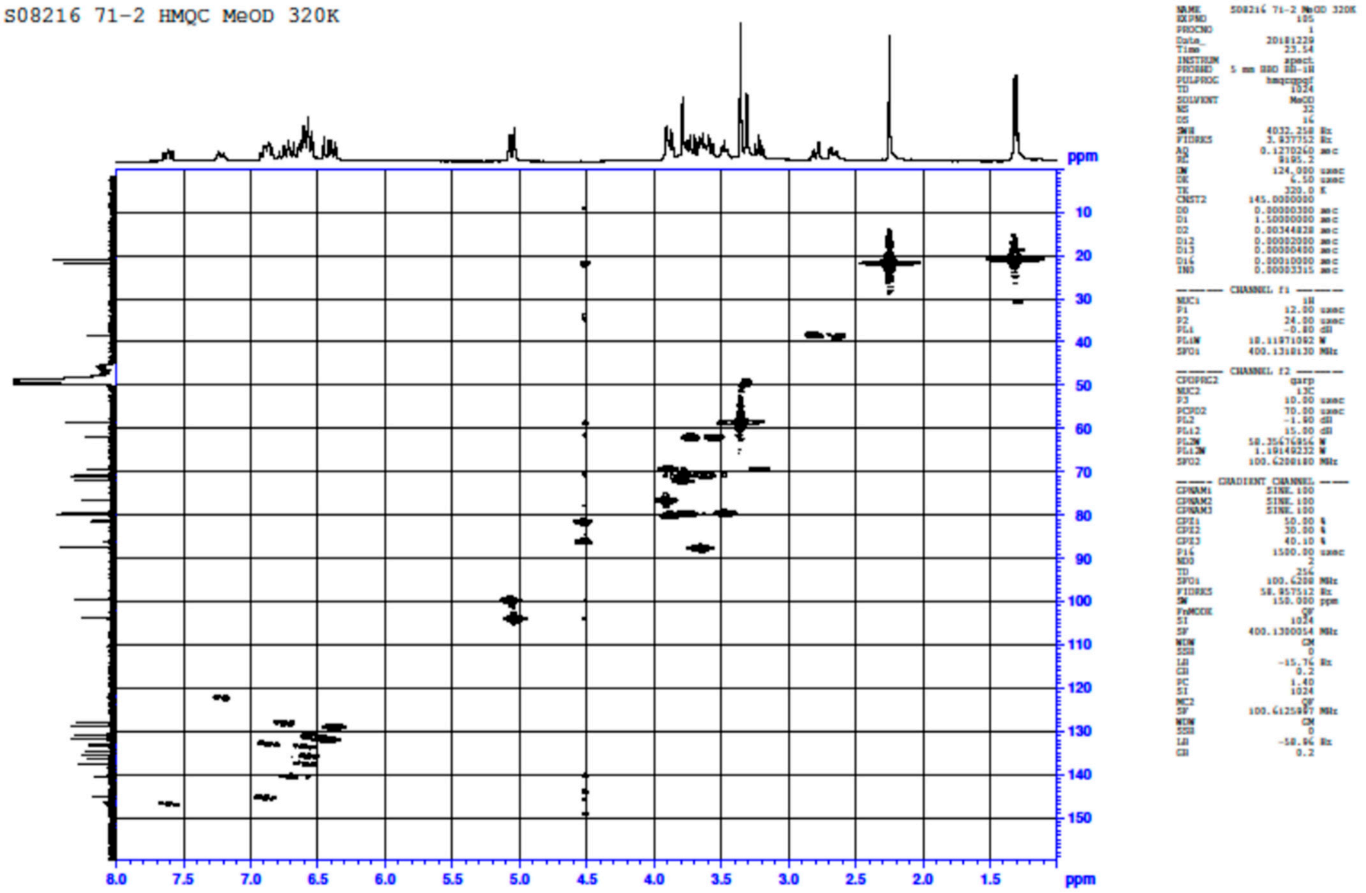

Figure S13. 2D ^{1}H-^{13}C HMQC spectrum of aurantoside L (**1**) (CD_3OD, 320 K).

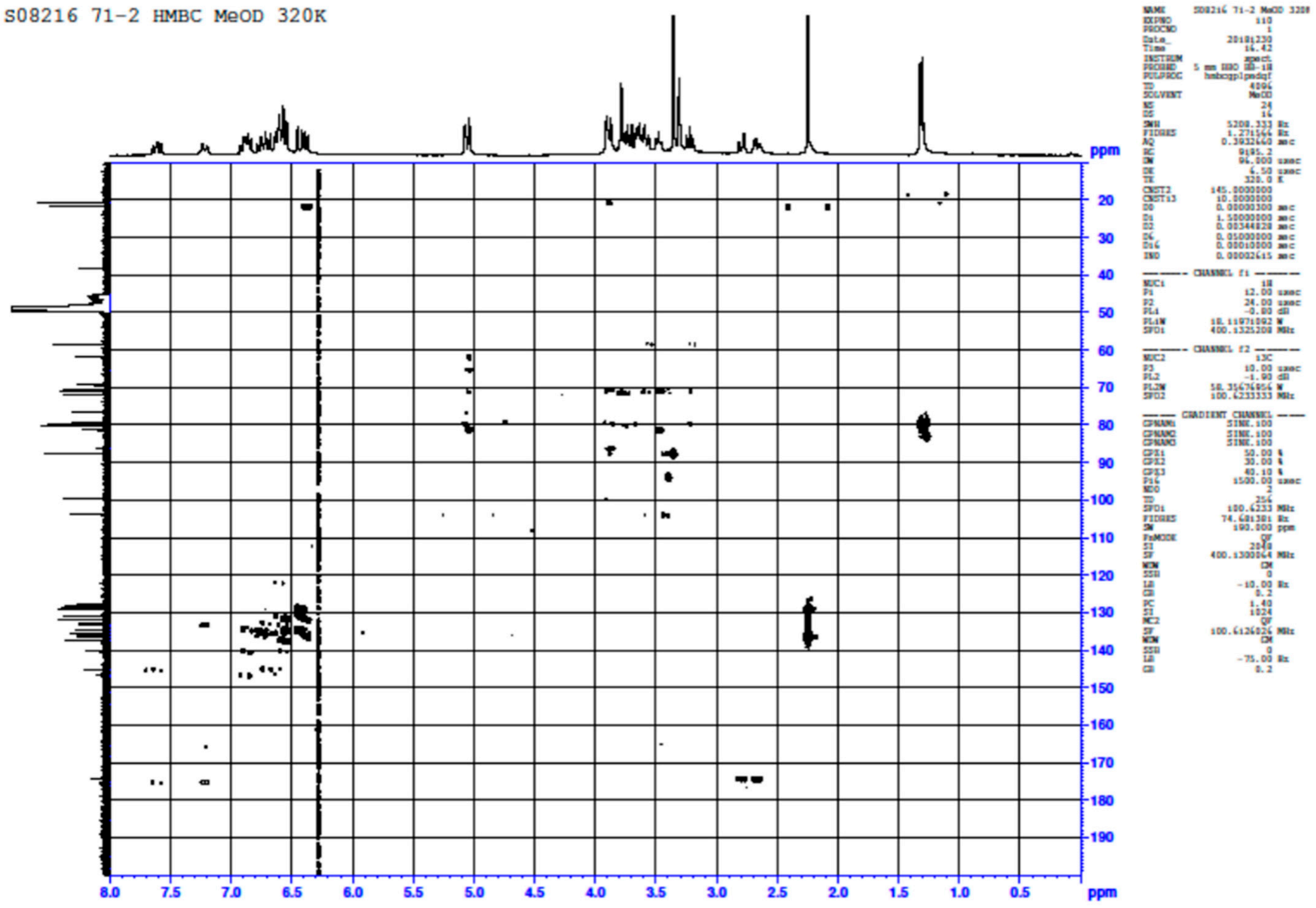

Figure S14. 2D ^{1}H-^{13}C HMBC spectrum of aurantoside L (**1**) (CD_3OD, 320 K).

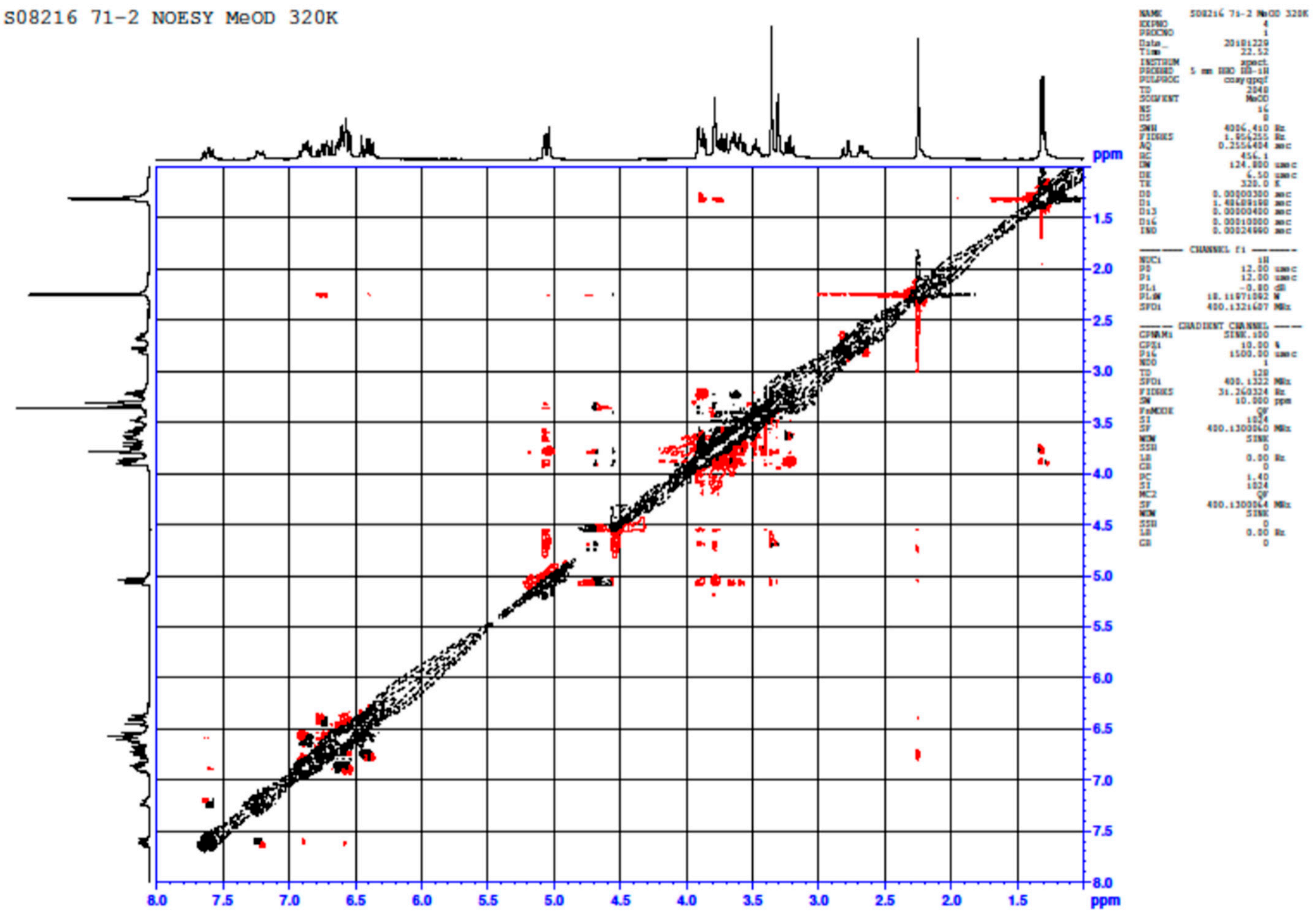

Figure S15. 2D ^1H-^1H NOESY spectrum of aurantoside L (**1**) (CD_3OD, 320 K).

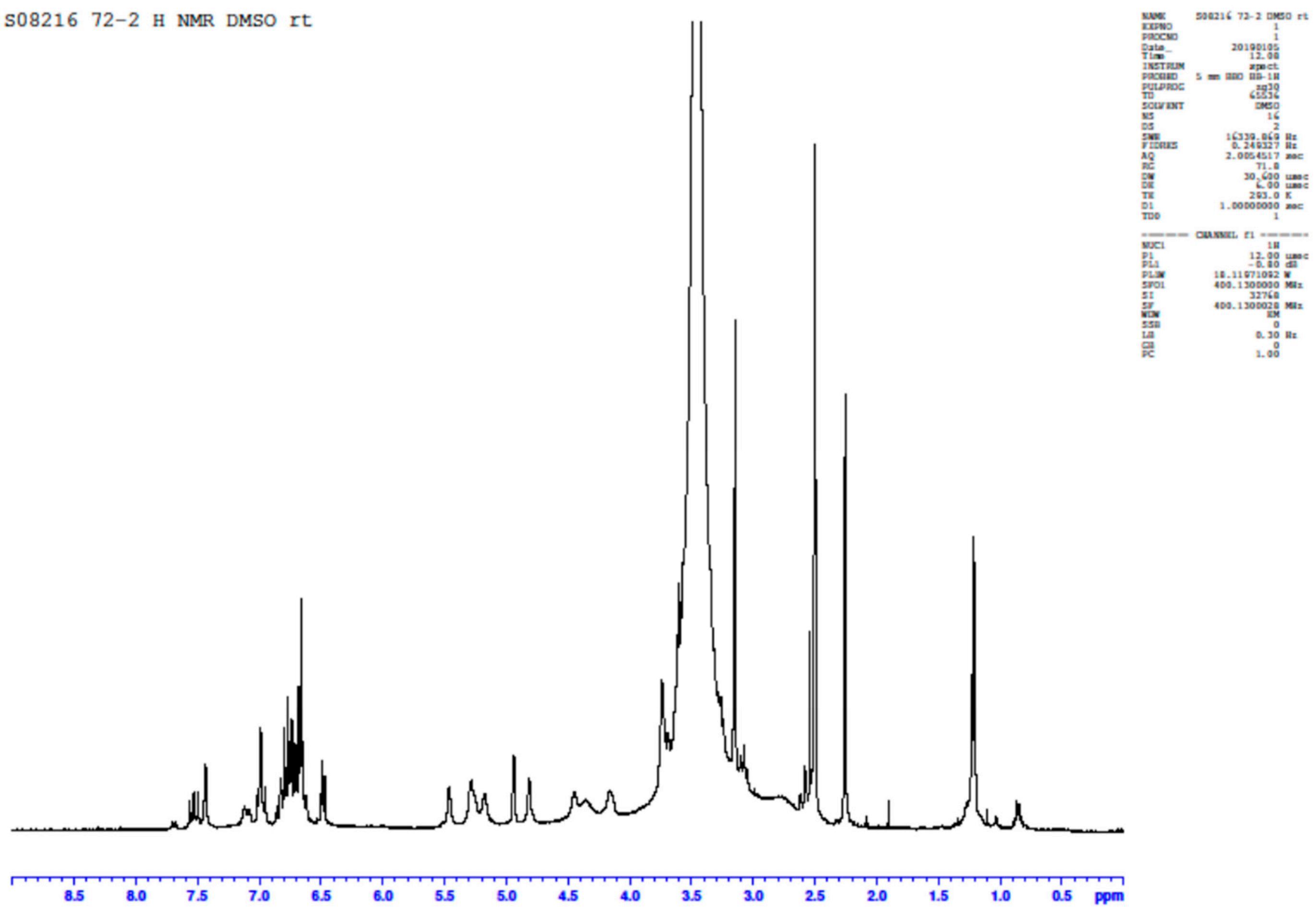

Figure S16. 1D ^{1}H NMR spectrum of aurantoside L (**1**) (CD_3COCD_3, 297 K).

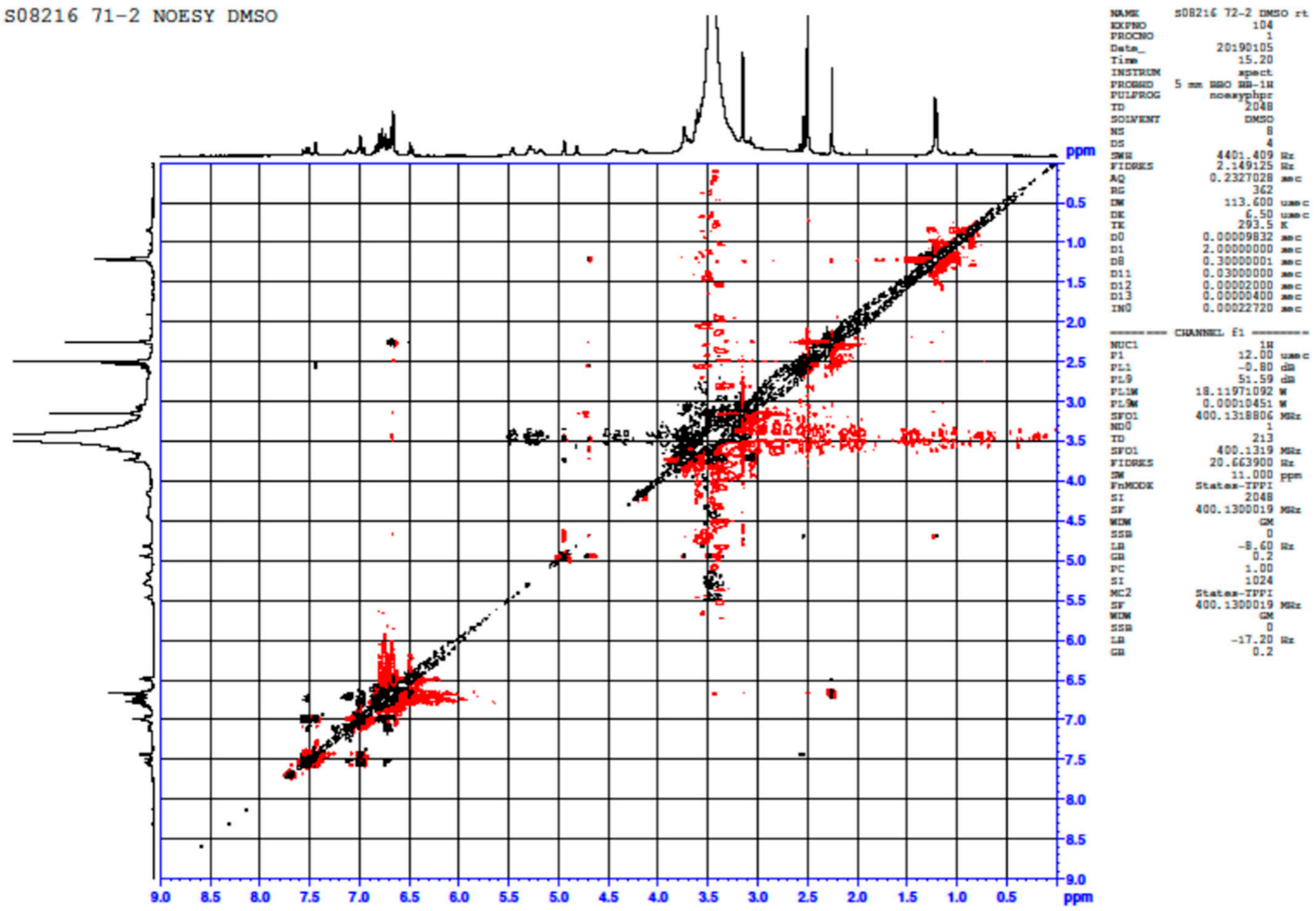

Figure S17. 2D ^1H-^1H NOESY spectrum of aurantoside L (**1**) (CD_3COCD_3, 297 K).

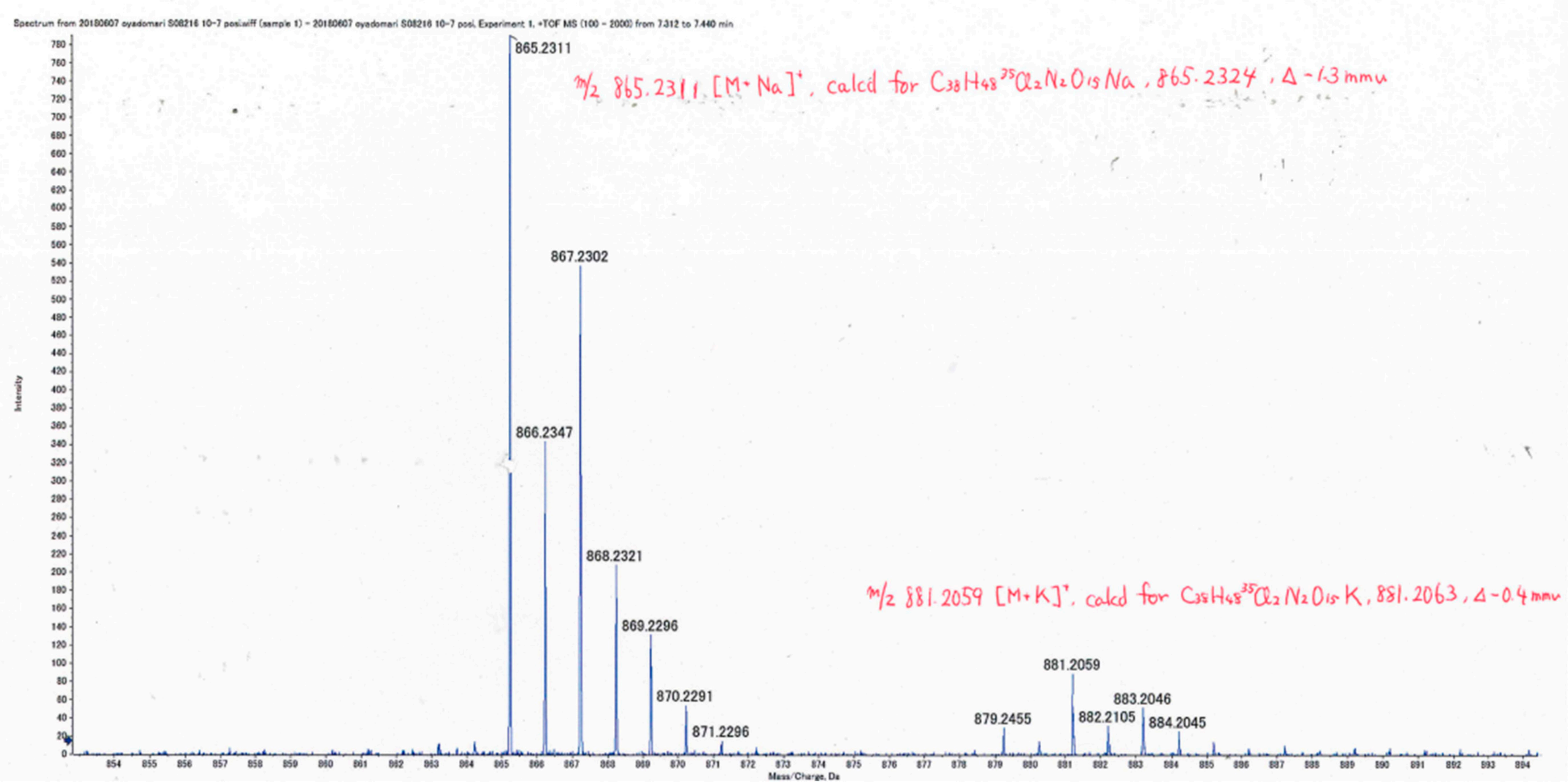

Figure S18. ESIMS of aurantoside L (**1**) (positive mode).

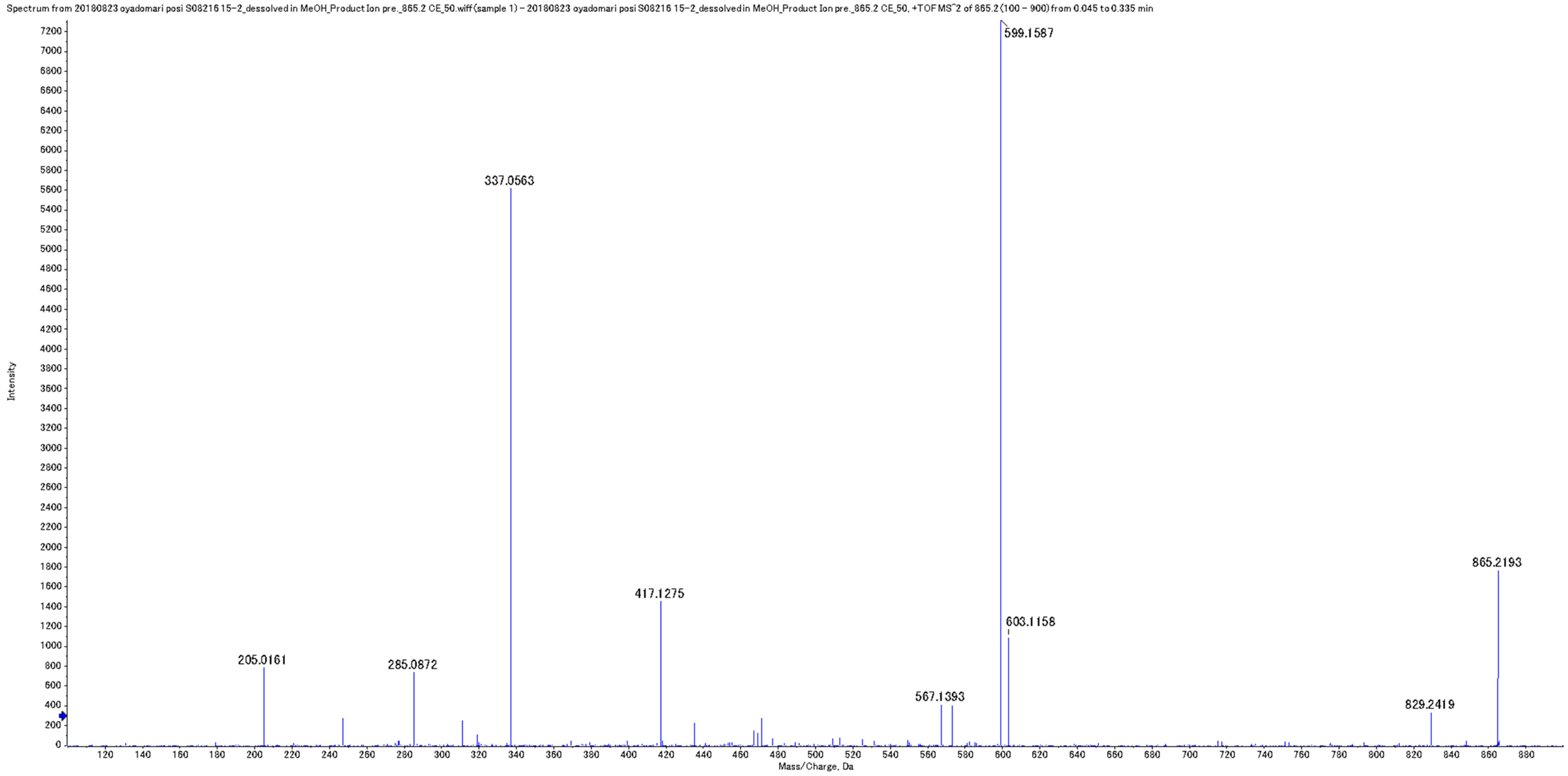

Figure S19. ESIMS/MS of aurantoside L (**1**) (positive mode, collision energy = 40 eV, precursor ion = *m*/*z* 865.2).
